# Opposing effects of Rho-associated coiled-coil kinase 1 (ROCK1) and ROCK2 in TGF-β-SMAD signaling

**DOI:** 10.1186/s12964-026-02722-5

**Published:** 2026-02-07

**Authors:** Yu Bai, Mohamad Moustafa Ali, Maarten van Dinther, Peter ten Dijke, Aristidis Moustakas, Anders Sundqvist, Carl-Henrik Heldin

**Affiliations:** 1 Department of Medical Biochemistry and Microbiology, Science for Life Laboratory, Biomedical Center, Uppsala University, Box 582, Uppsala, SE-75123 Sweden; 2 Present Address: Department of Immunology, Genetics and Pathology, Science for Life Laboratory, Uppsala University, Uppsala, SE-751 85 Sweden; 3 Department of Cell and Chemical Biology, Oncode Institute, Leiden University Medical Center, Leiden, The Netherlands; 4 Department of Pharmaceutical Biosciences, Uppsala University, Box 591, Uppsala, SE-751 24 Sweden

**Keywords:** Transforming growth factor-β (TGF-β), SMAD, Breast cancer, Rho-associated coiled-coil kinase 1 (ROCK1) and ROCK2, Cell proliferation, Invasion

## Abstract

**Background:**

Transforming growth factor-β (TGF-β) signals through type I and type II kinase-associated transmembrane receptors to activate both SMAD-dependent and SMAD-independent pathways, including the Rho/ROCK axis with its effectors ROCK1 and ROCK2. However, the role of ROCK isoforms in TGF-β-SMAD signaling in breast cancer cells, has not been elucidated.

**Methods:**

CAGA_12_-luciferase reporter assays were utilized to measure SMAD3/4-dependent transcription. Small-molecule kinase inhibitors and RNA interference-mediated gene silencing were provided for loss-of-function analyses. Immunoblotting, immunofluorescence staining, subcellular fractionation, in vitro kinase activity assay and mRNA expression assays were performed to gain mechanistic evidence. Mass spectrometry analysis coupled with co-immunoprecipitation assays examined the interaction between ROCK isoforms and other members of the TGF-ꞵ signaling pathway.

**Results:**

ROCK2 knockdown or treatment with the selective ROCK2 kinase inhibitor KD025 suppressed TGF-β-SMAD signaling, whereas ROCK1 knockdown produced the opposite effect. Overexpression studies revealed that ROCK1 inhibited, while ROCK2 enhanced, TGF-β-induced SMAD3/4-dependent transcriptional activity. These effects were reversed by expression of kinase-dead ROCK1 (K105R) or ROCK2 (K121D) mutants, and by treatment with ROCK inhibitors, demonstrating that the kinase activities of the two isoforms are required for their opposing functions. We further showed that ROCK1 and ROCK2 exhibit distinct subcellular localizations, and that SMAD3 interacts with ROCK1, but not with ROCK2. ROCK2 inhibition reduced phosphorylation at serine residues in the C-terminal (Ser423/425) and linker region (Ser204 and Ser208) of SMAD3, decreased nuclear accumulation of SMAD3 and SMAD4, and suppressed TGF-β-SMAD target gene expression. Functionally, ROCK1 depletion promoted cell proliferation and invasion in MDA-MB-231 cells, whereas ROCK2 depletion reduced proliferation and invasion.

**Conclusions:**

ROCK1 and ROCK2 exert opposing, kinase-dependent effects on TGF-β-SMAD signaling and differentially affect breast cancer cell behaviours.

**Supplementary Information:**

The online version contains supplementary material available at 10.1186/s12964-026-02722-5.

## Introduction

 Transforming growth factor-β (TGF-β) regulates a broad range of cellular processes, such as proliferation, migration, apoptosis and differentiation, and plays important roles in embryonic development, angiogenesis and wound healing, as well as in cancer progression [1]. TGF-β exerts its cellular effects through cell surface TGF-β type I (TβRI) and type II (TβRII) receptors, which are equipped with intrinsic kinase domains [2]. TGF-β first binds to TβRII, after which TβRI is recruited to the receptor complex and is activated by TβRII-mediated phosphorylation; activated TβRI phosphorylates SMAD2 and SMAD3, which form complexes with SMAD4. Heteromeric SMAD complexes translocate into the nucleus, where they cooperate with other transcription factors to either repress or activate the expression of specific genes [2]. SMAD2 and SMAD3 contain conserved N-terminal and C-terminal domains, i.e. Mad homology (MH)1 and MH2 domains, respectively, connected with linker regions containing a proline-tyrosine (PY)-motif [3]. The SMAD3 linker region contains four proline-directed kinase phosphorylation sites, i.e. Thr179, Ser204, Ser208, and Ser213, which can be phosphorylated by different kinases under different conditions. For example, cyclin- dependent kinase (CDK) 4 and CDK2 phosphorylate SMAD3 at Thr179 and Ser213 and inhibit its transcriptional activity and anti-proliferative effect [4]. TGF-β can also activate several non-SMAD signaling pathways, such as ERK1/2, JNK and p38 mitogen-activated protein (MAP)-kinases, phosphatidylinositol 3´-kinase (PI3K)-AKT, the tyrosine kinase SRC and Rho-associated coiled-coiled kinase (ROCK) [5, 6].

There are two ROCK isoforms encoded in the human genome, i.e. ROCK1 (also named ROCK I, ROKβ, Rho-kinase β, or p160ROCK) and ROCK2 (also termed ROCK II, ROKα, or Rho kinase), sharing 65% overall sequence identity and 92% sequence identity in the kinase domains [7]. The ROCK isoforms are the main downstream effectors of Rho small guanosine-5’-triphosphate (GTP)-binding proteins; both have a serine/threonine kinase domain in the N-terminal region, a pleckstrin homology (PH) domain in their C-terminal region and a coiled-coiled region with a Rho-binding domain in their central region [8, 9]. The C-terminal PH domain contains a cysteine-rich zinc finger-like domain that is involved in the stabilization of membrane binding of ROCK [10]. The ROCK isoforms regulate a wide spectrum of cellular processes, such as actin cytoskeleton organization, cell contraction, proliferation, migration, apoptosis and focal adhesion [11–14].

Breast cancer is a major cause of cancer-related death in women worldwide [15]. It is estimated that triple-negative breast cancer (TNBC) accounts for about 15% of all breast cancer cases. Because of the lack of receptor expression, TNBC patients respond poorly to hormonal or anti-human epidermal growth factor receptor (HER)2 targeted therapies, and have a worse prognosis as compared to patients with other subtypes of breast cancers [16]. Since the discovery of the ROCK isoforms, a number of ROCK kinase inhibitors have been developed and tested in preclinical and clinical trials, such as Fasudil (also termed as HA-1077) [17], Y-27,632 [18] and GSK429286A (also named as RHO-15) [19, 20] that inhibit both ROCK isoforms, and KD025 (also named as SLx-2119) which is a highly selective inhibitor of the ROCK2 kinase [21]. These inhibitors are promising candidates for the development of new anticancer therapeutics, although additional studies are needed to investigate their effects [9, 22, 23].

In this study, we report that ROCK1 and ROCK2 differentially affect TGF-β signaling, exerting opposite effects on TGF-β-induced SMAD3/4-dependent CAGA_12_-luciferase reporter activity, target gene expression, as well as on the proliferation and invasion of breast cancer cells. Furthermore, ROCK2 inhibition suppresses TGF-β-SMAD signaling and prevents nuclear translocation of SMAD3 and SMAD4.

## Materials and methods

### Cell culture

The human breast cancer cell lines MDA-MB-231, Hs578T, HCC38 and MCF-7, the human embryonic kidney cell line HEK293T, the human non-small cell lung cancer cell line A549, the human keratinocyte cell line HaCaT and the mouse mammary epithelial cell line NMuMG were cultured in Dulbecco´s Modified Eagle´s Medium (DMEM; Gibco, Life Technologies Ltd, Paisley, UK), supplemented with 10% fetal bovine serum (FBS) (Gibco, Life Technologies Ltd, Paisley, UK). The human breast cancer cell lines BT-549, HCC1954 and MDA-MB-453 were cultured in RPMI-1640 (Gibco, Life Technologies Ltd, Paisley, UK), supplemented with 10% FBS. The MDA-MB-231 HiBiT-3×FLAG-activin receptor-like kinase (ALK)5 (also termed TꞵRI) and HiBiT-3×FLAG-TꞵRII cell lines were established by the CRISPR-Cas9 technique, and cultured in DMEM with 10% FBS. The stable MDA-MB-231-pLKO, -shROCK1 and -shROCK2 cell lines were cultured in DMEM, supplemented with 10% FBS and 0.8 µg/ml puromycin (Sigma-Aldrich Sweden AB, Stockholm, Sweden). MCF A MII cells were cultured in DMEM/F12 (Gibco, Life Technologies Ltd, Paisley, UK), supplemented with 5% FBS, 20 ng/ml epidermal growth factor (EGF) (PeproTech, EC Ltd, London, UK), 200 mM L-glutamine (Thermo Fischer Scientific, Sweden), 100 ng/ml cholera toxin, 0.5 µg/ml hydrocortisone, 10 µg/ml insulin; the latter four reagents were from Sigma-Aldrich AB, Stockholm, Sweden. The MCF 10AMII-pLKO, -shROCK1 and -shROCK2 cells were maintained in the same medium as the WT cell line, supplemented with 0.5 µg/ml puromycin. The MCF A MII-CAGA-luc cells were also cultured in the same medium as the WT cell line, and supplemented with 1.0 µg/ml puromycin. All cell lines were incubated at 37°C and in 5% CO_2_ in a humidified incubator, and were confirmed to be free of mycoplasma using the Mycoplasma Checking kit (Eurofins Genomics, Sweden).

### Reagents, antibodies and plasmids

Recombinant human TGF-β1 (denoted TGF-β in this study) was purchased from PeproTech (EC Ltd, London, UK). The small molecule TβRI (ALK5) kinase inhibitor (SB505124, used at 2.5 µM) was obtained from Sigma-Aldrich AB, Stockholm, Sweden. The pan-ROCK kinase inhibitor (GSK429286A, used at 10 µM) and the highly selective ROCK2 kinase inhibitor (KD025, used at 5 µM) were obtained from Selleckchem, Houston, USA. Puromycin, used at a concentration of 0.5 or 0.8 µg/ml, was obtained from Sigma-Aldrich AB, Stockholm, Sweden. The antibodies used in this study are listed in Supplementary Table S1.

The On-target plus Non-Targeting Control siRNA (Cat no: D-001810-01-20), the ROCK1 On-target plus siRNA pool (Cat no: L-003536-00-0005) and the ROCK2 On-target plus siRNA pool (Cat no: L-004610-00-0005) were purchased from Dharmacon (Horizon Discovery, Cambridge, UK). The final concentration used was 25 nM and cells were transfected using SiLentFect (Bio-Rad Laboratories AB, Solna, Sweden) transfection reagent, according to the manufacturer’s instructions.

Plasmids for GFP-ROCK1 wild-type (WT) and GFP-ROCK2 WT were obtained from Addgene (Watertown, MA, USA); plasmids for the kinase-dead mutants ROCK1 (K105R) and ROCK2 (K121R) [13, 24, 25] were constructed using a site-directed mutagenesis kit (QuikChange Lightning, Agilent Technologies, Sweden). The primer sequences used to generate different mutants are shown in Supplementary Table S2. All the plasmid sequences were confirmed by DNA sequencing (Eurofins Genomics, Ebersberg, Germany).

### Lentiviral transduction

MDA-MB-231 and MCF10A MII cells were infected with lentiviruses encoding shRNA sequences against human ROCK1 (TRCN0000002159; TRCN0000195202) or human ROCK2 (TRCN0000000977; TRCN0000000978), selected from the MISSION shRNA library (Sigma-Aldrich, Chemie B.V. Netherlands). An empty pLKO vector was used as a control. Lentiviral virus transduction was performed overnight, and the infected cells were selected using culture medium, supplemented with puromycin at 0.5 or 0.8 µg/ml.

The generation of the MCF10A MII-CAGA-luc cell line was performed as described in Ali et al. (*Cell Death & Disease*, in press, 2026). Briefly, lentiviral vectors containing the CAGA_12_-luciferase reporter within the pGL4-MLP backbone were co-transfected into HEK293T cells with pCMV-VSV-G-RSV-Rev and pCAG-HIVgp to produce lentiviral particles. Viral supernatants were collected and used to transduce MCF10A MII cells, followed by selection with 1 µg/mL puromycin to establish a stable reporter line.

### Quantitative real-time RT-PCR

Total RNA was isolated by RNA Purification Kit (Norgen Biotek Corp, Canada) and cDNA was synthesized with a High Capacity cDNA Reverse Transcription Kit (Applied Biosystems, Life Technologies, Ltd, Paisley, UK), according to the manufacturer’s specifications. Real-time qPCR was performed by using 2× qPCR SyGreen Mix (PCR Biosystems, London, UK) and CFX96 real-time PCR detection system (Bio-Rad Laboratories AB, Solna, Sweden); the values of expression were normalized to *GAPDH*. The primer sequences used in this study are shown in Supplementary Table S3. Experiments were performed at least three times.

### Luciferase assay

Cells were transiently transfected with the CAGA_12_-luciferase reporter or with pCMV-β-galactosidase (β-gal) as a control for transfection efficiency and for normalization, and were incubated for 24 h. Additional siRNAs or other constructs were co-transfected, as indicated. Cells were then stimulated or not with TGF-β (1 ng/ml) for another 24 h, after which they were subjected to Firefly Luciferase Assay Kit 2.0 (BioNordika, Solna, Sweden) to measure the luciferase activity according to the protocols of the manufacturer. Experiments were performed at least three times.

### Immunoprecipitation and Immunoblotting

Cells were cultured, starved and stimulated or not with TGF-β, at the indicated concentrations, for different time periods. Then the cells were washed with ice-cold phosphate buffered saline (PBS) two times and lysed in an immunoprecipitation (IP) lysis buffer (1% Triton X-100, 50 mM Tris-HCl, pH 7.4, 150 mM NaCl, 1 mM EDTA), supplemented with protease and phosphatase inhibitors (Roche Diagnostics, Scandinavia AB, Bromma, Sweden), and incubated on ice for 15 min. In certain experiments, the Anti-FLAG M2 affinity gel (Merck Life Science AB, Solna, Sweden) was used. After washing twice in PBS, cell lysates were added to the agarose beads and incubated at 4 °C overnight. In other experiments, soluble antibodies were incubated with ProteinA Dynabeads (Invitrogen, Life Technologies, Ltd, Paisley, UK) for 6 h at 4 °C, then incubated with cell lysates overnight at 4 °C. After washing the beads with an ice-cold lysis buffer two times and once with PBS, the bound proteins were eluted in 2× SDS sample buffer (5% sodium dodecyl sulphate (SDS), 25% glycerol, 150 mM Tris-HCl, pH 6.8, 0.01% bromophenol blue), and boiled for 3 min at 95 °C. Then the samples were centrifuged at 6,000×g for 30 s, after which the supernatants were subjected to SDS-polyacrylamide gel electrophoresis (SDS-PAGE) and transferred to a nitrocellulose membrane (Amersham Protran, GE Healthcare Life Science). For immunoblotting (IB), the membranes were blocked with 5% bovine serum albumin (BSA) diluted in TBS (50 mM Tris-HCl, pH 7.5, 150 mM NaCl) with 0.05% Tween-20 (TBST), and incubated with primary antibodies overnight at 4 °C. The next day, the membranes were incubated with secondary antibodies for 1 h at room temperature and then washed three times with TBST. The primary and secondary antibodies were used according to the supplier’s recommendations. Immuno-complexes were detected with the Immobilon Western kit (Merck Millipore, Solna, Sweden) on a charge-coupled device (CCD) camera (Bio-Rad Laboratories AB, Solna, Sweden). Experiments were performed three times, and representative results are shown.

### Mass spectrometry analysis

Total cell lysates of MDA-MB-231-HiBiT-3×FLAG-TꞵRI cells, after treatment or not with TGF-β (5 ng/ml) for 15 or 60 min, were subjected to immunoprecipitation with the Anti-FLAG M2 affinity gel (A2220, Merck Life Science AB, Solna, Sweden). After washing 3 times in IP lysis buffer (1.0% Triton X-100, 50 mM Tris-HCl, pH 7.5, 150 mM NaCl, 1 mM EDTA, 10% glycerol), proteins bound to beads were subjected to mass spectrometry analysis at the Clinical Proteomics Mass Spectrometry Facility, Science for Life Laboratory, Karolinska Institutet, Sweden.

Briefly, on-bead reduction, alkylation and digestion by trypsin (sequencing grade modified, Pierce, Thermo Fischer Scientific, Sweden) were performed, followed by SP3 peptide clean-up of the resulting supernatant [26]. Each sample was separated using a Thermo Scientific Dionex nano LC-system in a 3 h 5–40% acetonitrile gradient coupled to Thermo Scientific High Field QExactive. The software Proteome Discoverer vs. 1.4 including Sequest-Percolator for improved identification was used to search the *Homo sapiens* Uniprot database for protein identification, and filtered to a 1% false discovery rate (FDR) cut-off.

### Phalloidin staining

NMuMG cells were seeded onto 8-well culture slides (#354118, Falcon, Life Sciences, USA), and cultured in DMEM, supplemented with 10% FBS overnight, then washed with PBS two times, fixed with 3.5% paraformaldehyde (PFA) for 10 min, and permeabilized with 0.25% Triton X-100 for 10 min at room temperature. Cells were then washed twice in PBS and incubated with phalloidin (ab235137, Abcam, Cambridge, UK; diluted 1:1000 in PBS) for 15 min. Thereafter, the cells were washed with PBS three times and mounted in Fluoromount™ Aqueous Mounting Medium with 4’,6-diamidino-2-phenylindole (DAPI; F4680, Sigma-Aldrich AB, Stockholm, Sweden). Capture of images was performed by a Zeiss LCM700 inverted confocal microscope (Biovis, Uppsala University, Sweden). Images were exported as merged TIFF files with 8-bit resolution and individual channels were separated in Fiji (ImageJ) software; brightness was adjusted equally for all images within each experiment. Experiments were performed twice, and representative images are shown.

### Immunofluorescence staining

MDA-MB-231 and NMuMG cells were seeded onto 8-well culture slides (#354118, Falcon, Life Sciences, USA) and cultured in DMEM, 10% FBS overnight, then washed with PBS two times, followed by fixation with 3.5% PFA for 10 min and permeabilization in 0.25% Triton X-100 for 10 min at room temperature. The slides were then incubated with 1% BSA in PBS for 1 h at room temperature, and then incubated with indicated antibodies overnight. Cells were washed two times with PBS, and then incubated with a secondary antibody (Alexa Fluor 488, Alexa Fluor 555, and Alexa Fluor 647 diluted 1:500 in PBS) for 1 h at room temperature. The cells were then washed with PBS 3 times and mounted in Fluoromount™ Aqueous Mounting Medium with DAPI (F4680, Sigma-Aldrich AB, Stockholm, Sweden). Images were acquired using a 90i Nikon microscope (IMBIM, Uppsala University, Sweden) and a Zeiss LCM700 inverted confocal microscope (BioVis, Uppsala University, Sweden). Experiments were performed twice and representative images are shown.

### Subcellular fractionation

MDA-MB-231 cells, with or without knockdown of ROCK isoforms, were starved overnight, then incubated or not with TGF-β for 6 h, then harvested and lysed for separation of the nuclear and cytoplasmic fractions. Briefly, cells were rinsed with ice-cold PBS twice, scraped in 1 ml PBS, and centrifuged at 4 °C for 5 min at 10,000 rpm. The cell pellet was resuspended in lysis buffer (1% Triton X-100, 10 mM MES pH 6.2, 10 mM NaCl, 1.5 mM MgCl_2_, 1 mM EDTA, supplemented with protease inhibitors), and then incubated on ice for 15 min. After centrifugation of the lysates at 3,500 rpm for 12 min at 4 °C, the supernatants were saved as the cytoplasmic fractions. The pellets were resuspended in washing buffer (10 mM MES, pH 6.2, 10 mM NaCl, 1.5 mM MgCl_2_, 1 mM EDTA), and centrifuged at 3,500 rpm for 5 min at 4 °C; this step was repeated three times. The pellets were next resuspended in nuclear extraction buffer (0.5% Triton X-100, 25 mM Tris-HCl, pH 7.5, 1 mM EDTA, 0.5 M NaCl, supplemented with protease inhibitors), and incubated under rotation for 20 min 4 °C, followed by centrifugation at 13,000 rpm for 15 min; the supernatant was saved as the nuclear fraction. Proteins were then quantified and samples were analyzed by immunoblotting. Experiments were performed three times, and representative results are shown.

### In vitro phosphorylation assay

The kinase activities of ROCK isoforms were measured using the ROCK1 Kinase Enzyme System (V3411, Promega Biotech AB, Sweden), the ROCK2 Kinase Enzyme System (V4044, Promega Biotech AB, Sweden), and the ADP-Glo™ Kinase Assay Kit (V9101, Promega Biotech AB, Sweden) to assess the possibility that SMAD3 is phosphorylated by ROCK isoforms. The reaction buffer contained 40 mM Tris-HCl (pH 7.5), 20 mM MgCl₂, 0.1 mg/mL BSA and 50 µM dithiothreitol (DTT). Each reaction contained 25 µM ATP, 10 ng ROCK1 or ROCK2 kinase, and the S6K peptide (0.2 mg/ml) as a positive control substrate. The recombinant human-derived SMAD3 (HY-P71323, MedChemExpress, Sollentuna, Sweden) was tested at 1.0 µM. Reactions were incubated at room temperature for 30 min, and ATP consumption was subsequently quantified according to the manufacturer’s instructions. The chemiluminescent signal from the final luciferase-based reaction was measured using a plate-reading luminometer (PerkinElmer, USA). ROCK-mediated SMAD3 phosphorylation was calculated as (ROCK + SMAD3 + ATP) - (ROCK + ATP) - (SMAD3 + ATP). Experiments were performed three times.

### Cell proliferation assay

MDA-MB-231-pLKO cells, or cells stably transfected with shROCK1 and shROCK2, were counted using a LUNA™ Cell Counting slides (L12001, Logos Biosystems, BioNordika, Sweden), then seeded in 48-well plates with 5,000 cells/well and starved in DMEM, supplemented with 0.2% FBS overnight. Cells were then treated or not with TGF-β (5 ng/ml). After 48 h, the cells were washed twice with PBS and subjected to the CyQUANT^®^ GR Cell proliferation assay (C7026, Invitrogen, Life Technologies, Ltd, Paisley, UK), used according to the manufacturer’s instructions. The CyQuant GR dye solution was added to each well and samples were incubated for 5 min at room temperature in darkness. After excitation at 480 nm, fluorescent emission at 520 nm, was then determined by a plate reader (PerkinElmer, USA). Experiments were performed at least three times.

### Transwell invasion assay

Cell invasion was analyzed using transwell plates with 6.5 mm diameter and 8 μm pore filters (#351152, Corning, NY, USA). Inserts were coated with 300 µg/ml Matrigel (#356234, Corning, NY, USA) by incubation at 37 °C for 1 h. MDA-MB-231 cells (5 × 10^4^), transfected with control, ROCK1 or ROCK2 siRNAs, were seeded in the upper chamber in serum-free DMEM, and DMEM supplemented with 10% FBS was placed in the lower chamber. Following 24 h, the inserts were fixed in ice-cold methanol. Cell nuclei were stained with DAPI diluted at 1:1,000 in PBS. Cell invasion capacity was measured by counting the nuclei of cells that had migrated through the filter pores towards the chemoattractant (10% FBS). For quantification, 20 pictures of each insert were taken at 20× magnification and nuclei were counted using the Fiji (ImageJ) software. Experiments were performed three times.

### Pathway enrichment analyses

The normalized peptide-spectrum match (PSM) values of the significantly enriched proteins were used for sample clustering and generation of heatmaps by utilizing an online platform (bioinformatics.com.cn) for data analysis and visualization [27]. Analyses and visualization of significantly enriched proteins were performed using the Enrich tool ShinyGo 0.85 [28] to query the Gene Ontology biological process database.

### Correlation between ROCK isoform expression and patient survival

Relapse-free survival (RFS) of breast cancer patients was predicted using the Kaplan-Meier Plotter database (https://kmplot.com/analysis/index.php?p=service). The analysis was performed using Affymetrix microarray probe IDs for ROCK1 (235854_x_at) and ROCK2 (202762_at). Samples were stratified, and compared using median cut-off, and the p-value was calculated by log-Rank test.

### Statistical analysis

Statistical analyses were performed with GraphPad Prism software. The statistical significance of two-group comparison’s differences was determined by two-tailed unpaired Student’s t-tests. The statistical significance of multiple comparisons was assessed by two-way ANOVA followed by multiple-comparison tests. Additional statistical methods are included in the figure legends. *, *p* < 0.05 was considered as statistical significant; **, *p* < 0.01; ***; *p* < 0.001; ****, *p* < 0.0001; ns, not significant difference.

## Results

### ROCK1 interacts with TβRI

To identify novel proteins that interact with TGF-ꞵ receptors, we introduced sequences encoding HiBiT and 3×FLAG tags in the *TGFβR1* gene, and the *TGFβR2* gene in MDA-MB-231 cells, by CRISPR-Cas9 technology, to establish the MDA-MB-231-HiBiT-3×FLAG-TβRI and the MDA-MB-231-HiBiT-3×FLAG-TβRII cell lines. We confirmed that the TGF-β-induced SMAD C-terminal and p38 MAPK phosphorylation levels (Supplementary Figure S1A), and TGF-β target gene expression (Supplementary Figure S1B), were similar in MDA-MB-231 WT and in the modified cell lines. For a proteomic TꞵRI-interaction screen, the MDA-MB-231-HiBiT-3×FLAG-TꞵRI cells were treated or not with TGF-β (5 ng/ml) for 15 min and 60 min. Cell lysates were then subjected to immunoprecipitation using beads with immobilized FLAG antibodies; after validation that TꞵRI was immunoprecipitated and the positive control TꞵRII was coprecipitated (Supplementary Figure S1C), bound proteins, in triplicates, were then analysed by mass spectrometry (SciLifeLab, Karolinska Institute, Stockholm, Sweden).

**Fig. 1:**
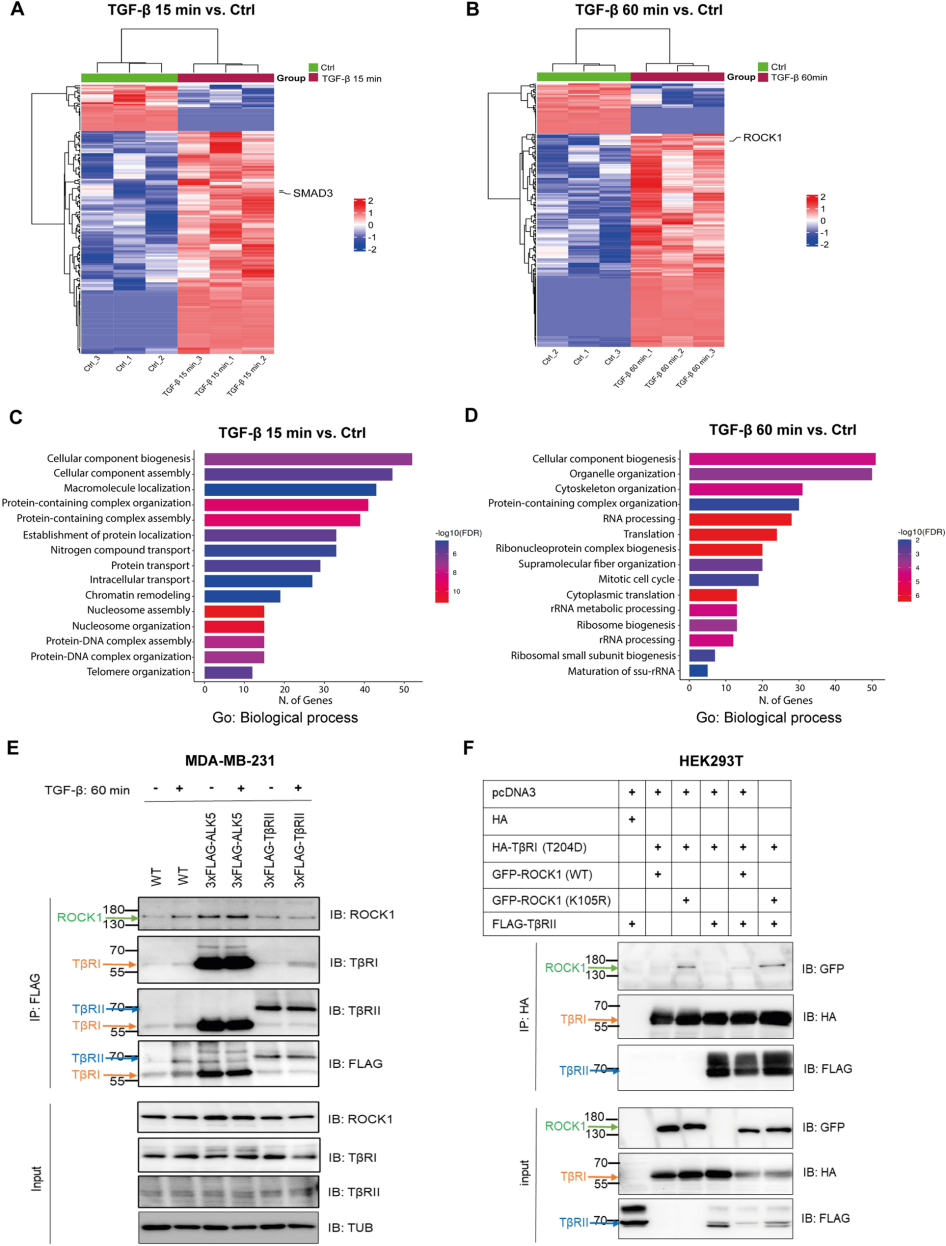
ROCK1 interacts with the TGF-β type I receptor, as identified by a mass spectrometry screen and co-immunoprecipitation. **A**, **B** Heatmaps of mass-spectrometry analysis of triplicate samples per biological condition (TGF-β treatment for 0 min, 15 min or 60 min). **C**, **D** Gene ontology (Go): biological process enrichment analysis of the significantly enriched proteins that interact with TβRI at 15 min and 60 min of TGF-β stimulation, by ShinyGO 0.85. **E** Co-immunoprecipitation (IP) of TβRI and ROCK1. MDA-MB-231-HiBiT-3×FLAG-TβRII and WT MDA-MB-231 cells, used as controls, were serum-starved and then stimulated or not with TGF-β (5 ng/ml) for 1 h. Cell lysates were then subjected to IP with a FLAG antibody, and immunoblotting (IB) with the antibodies indicated in the figure. **F** Co-immunoprecipitation of TβRI and ROCK1 after overexpression by transfection. HEK293T cells were transfected with HA-TβRI (T204D), FLAG-TβRII, GFP-ROCK1 and GFP-ROCK1 (K105R) plasmids, cell lysates were then subjected to IP with an HA antibody, followed by IB with the indicated antibodies

Following peptide retrieval and database query, we filtered the identified proteins based on a minimal false discovery rate (q-value) ≤ 0.01. We calculated the relative abundance of each protein using peptide spectrum match (PSM) values, which were converted to log10 values to calculate the fold enrichment and statistical significance. We generated heatmaps based on quantified PSM values of each biological replicate, showing the significantly bound proteins to TꞵRI before and after TGF-ꞵ stimulation for 15 min and 60 min (Fig. 1A and B). Furthermore, we performed pathway enrichment analysis of the significantly enriched proteins obtained in each condition (15–60 min stimulation vs. control) utilizing the Gene Ontology (GO) database. The analysis indicated that the enriched proteins take part in several pathways in biological processes (Fig. 1C and D), including cytoskeleton organization, cellular component biogenesis and cytoplasmic translation. Our analysis revealed that TβRI interacted with ROCK1, and that the interaction was enhanced after TGF-β stimulation for 60 min (FC = 1.3908; p-value = 0.010016). We decided to explore further the involvement of ROCK1, and also of ROCK2, in TGF-ꞵ signaling.

To verify the interaction between TβRI and ROCK1, we performed a co-immunoprecipitation (IP) assay using MDA-MB-231-HiBiT-3×FLAG-TꞵRI cells; MDA-MB-231 WT cells and MDA-MB-231-HiBiT-3×FLAG-TβRII cells served as negative controls. Immunoprecipitation with a FLAG antibody followed by immunoblotting (IB) with a ROCK1 antibody, revealed an interaction between TβRI and ROCK1, with and without stimulation with TGF-β (Fig. 1E). In contrast, ROCK2 was not detected in the TβRI precipitate under the same experimental conditions, indicating isoform-specific interaction (data not shown). Moreover, we investigated by co-immunoprecipitation, whether TβRI and ROCK1 interacted in HEK293T cells after transfection of plasmids encoding TβR1, ROCK1 and its kinase-dead mutant, ROCK1 (K105R). We found that TβRI interacted with ROCK1 and that the interaction was stronger with the kinase-dead ROCK1 mutant (Fig. 1F).

### ROCK isoforms have opposite effects on TGF-β-induced SMAD3/4-driven transcriptional activity

**Fig. 2:**
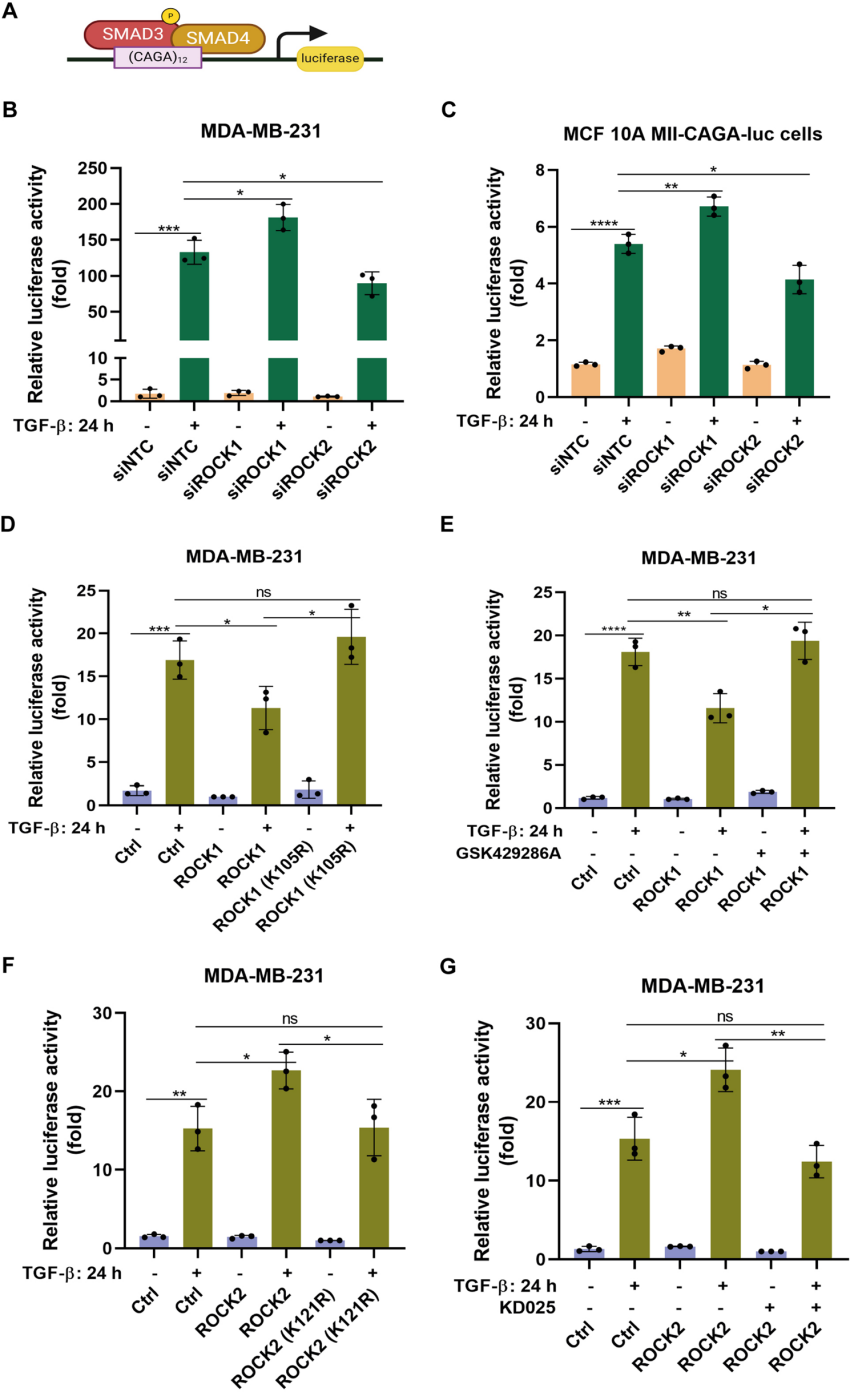
ROCK1 and ROCK2 have opposite effects on TGF-β-induced activation of the CAGA12-Luc reporter. **A** Schematic representation of the luciferase reporter to examine the transcriptional activity of SMAD3. **B**, **C** MDA-MB-231 cells (**B**) and MCF10A MII-CAGA-luc stable cells (**C**), were transfected with siControl (siNTC), siROCK1, siROCK2, and co-transfected with CAGA12-luc and β-gal plasmids. **D-G** MDA-MB-231 cells, were transfected with CAGA12-luc and β-gal plasmids, and with plasmids for GFP-ROCK1 WT and a kinase-dead ROCK1 mutant (K105R) (**D**) treated with a pan-ROCK kinase inhibitor GSK429286A (**E**) transfected with GFP-ROCK2 WT and kinase-dead ROCK2 mutants (K121R) (**F**), or treated with the highly selective ROCK2 kinase inhibitor KD025 (**G**). Cells were then starved and stimulated or not with TGF-β (1 ng/ml) for 24 h. The luciferase activities were measured by a Firefly Luciferase Assay Kit. Data are presented in panels **B-G** as mean ± standard deviation (SD) of three independent experiments; statistical significance was assessed by two-tailed unpaired Student’s t-tests. ns, not significant difference; *, *p*<0.05; **, *p*< 0.01; ***, *p*< 0.001; ****, *p*< 0.0001

In order to explore a possible cross-talk between non-SMAD and SMAD-dependent pathways, we investigated the effect of the two ROCK isoforms (ROCK1 and ROCK2), on TGF-β-SMAD signaling. We used the CAGA_12_-luciferase reporter, which responds to activated SMAD3 and SMAD4, to monitor the activity of the TGF-β-SMAD signaling pathway (Fig. 2A) [29]. Transient silencing of individual ROCK isoforms using specific small interfering RNA (siRNA) against ROCK1 or ROCK2 in MDA-MB-231 cells, revealed that depletion of ROCK1 increased TGF-β-induced expression of CAGA_12_-luciferase activity, whereas, interestingly, depletion of ROCK2 suppressed CAGA_12_-luciferase activity (Fig. 2B). Similar results were also obtained in MCF10A MII-CAGA-luc cells, which stably express CAGA_12_-luciferase reporter (Fig. 2C). Moreover, ROCK1 knockdown by short hairpin RNA (shRNA) in MDA-MB-231-shROCK1 (Supplementary Figure S2A) and in MCF10A MII-shROCK1 (Supplementary Figure S2B) cell lines, enhanced TGF-β induced expression of CAGA_12_-luciferase activity. In contrast, depletion of ROCK2 in MDA-MB-231-shROCK2 cells (Supplementary Figure S2C) and in MCF10A MII-shROCK2 cells (Supplementary Figure S2D), reduced CAGA_12_-luciferase activity.

### Kinase activity of ROCK isoforms is needed for the regulation of SMAD3/4-dependent transcription

We found that ROCK1 overexpression significantly reduced TGF-β-induced CAGA_12_-luciferase activity in MDA-MB-231 cells (Fig. 2D and E), whereas expression of a kinase-dead ROCK1 mutant (K105R) or treatment with a ROCK inhibitor, GSK429286A, which inhibits both ROCK1 and ROCK2, reversed the inhibitory effect, suggesting that ROCK1 overexpression inhibits TGF-β-induced CAGA_12_-luc reporter expression in a kinase-dependent manner. In contrast, overexpression of ROCK2 enhanced CAGA_12_-luciferase activity in MDA-MB-231 cells (Fig. 2F and G), which was reversed by expression of a kinase-dead ROCK2 mutant (K121R) or treatment with the highly selective ROCK2 isoform inhibitor KD025. These observations support the notion that the kinase activities of ROCK1 and ROCK2 are required for their opposite effects on kinases to modulate SMAD3/4 dependent transcriptional activation.

### Differential effects of ROCK1 and ROCK2 in TGF-β-SMAD signaling

**Fig. 3:**
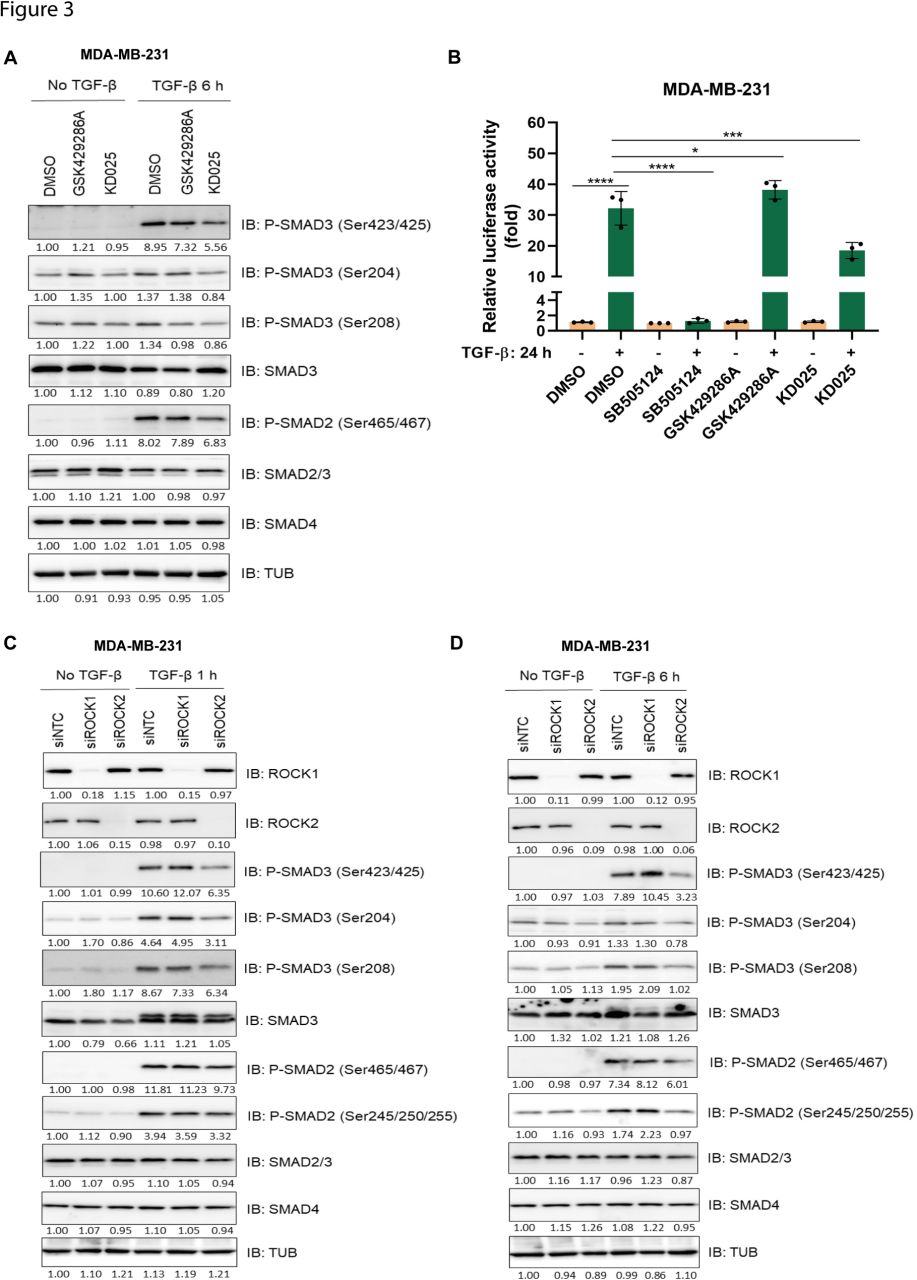
ROCK2 inhibition suppresses TGF-β-SMAD activity. **A** MDA-MB-231 cells were starved in DMEM, supplemented with 0.2% FBS, treated or not with KD025 (5 μM) and TGF-β (5 ng/ml) for 6 h. Total cell lysates were subjected to IB with the indicated antibodies. The intensities of bands were quantified and normalized to the DMSO condition set to 1.00, and further normalized to Tubulin (TUB), by Image Lab software. **B** MDA-MB-231 cells were starved in DMEM, supplemented with 0.2% FBS, treated or not with SB505124 (2.5 μM), GSK429286A (10 μM), KD025 (5 μM) and TGF-β (1 ng/ml) for 24 h. The luciferase activities were measured by a Firefly Luciferase Assay Kit. Data are presented in panel B as mean ± SD of three independent experiments; statistical significance was assessed by two-tailed unpaired Student’s t-tests. *, *p*<0.05; ***, *p*< 0.001; ****, *p*< 0.0001. **C**, **D** MDA-MB-231 cells transfected with siControl (siNTC), siROCK1, siROCK2, were starved in 0.2% FBS, DMEM, then incubated with or without TGF-β (5 ng/ml) for 1 h (**C**), or 6 h (**D**). Total cell lysates were subjected to IB with the indicated antibodies. The band intensities were quantified and normalized to the siNTC (No TGF-ꞵ) condition set to 1.00, and further normalized to Tubulin (TUB), by Image Lab software.

**Fig. 4:**
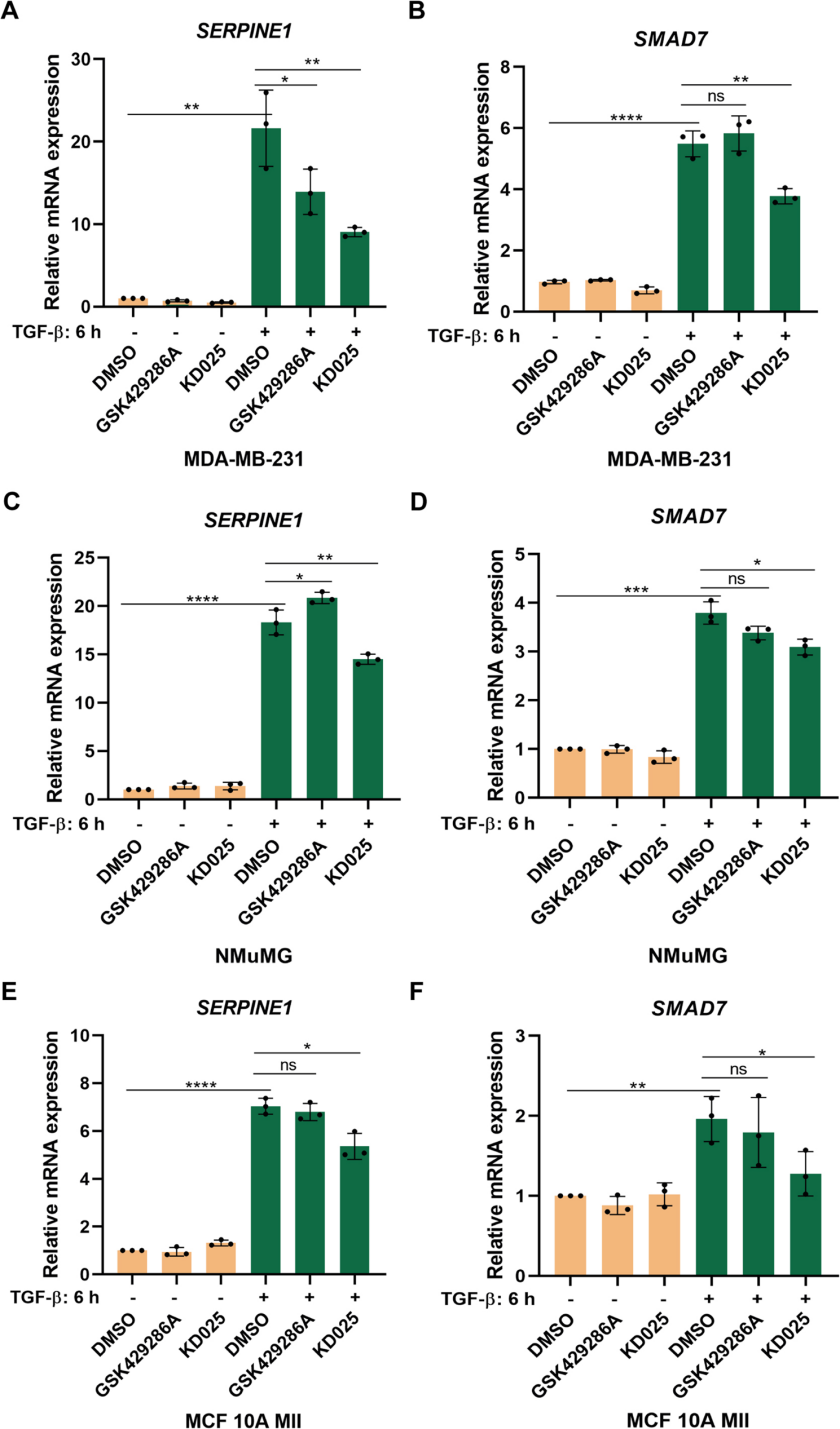
ROCK2 inhibition suppresses TGF-β-SMAD target gene expression. MDA-MB-231 cells (**A**, **B**), NMuMG cells (**C**, **D**), MCF 10A MII cells (**E**, **F**) were starved in medium, supplemented with 0.2% FBS, and treated or not with GSK429286A (10 μM), KD025 (5 μM) and TGF**-**β (5 ng/ml) for 6 h. Expression of SERPINE1 (**A**, **C**, **E**) and SMAD7 (**B**, **D**, **F**) was examined by RT-qPCR and normalized to GAPDH. Data are presented in panels **A-F** as mean ± SD of three independent experiments; statistical significance was assessed by two-tailed unpaired Student’s t-tests. ns, not significant difference; *, *p*<0.05; **, *p*< 0.01; ***, *p*< 0.001; ****, *p*< 0.0001.

To further elucidate the effects of ROCK isoforms in TGF-β-SMAD signaling, we compared the effects of the ROCK1/ROCK2 inhibitor GSK429286A with a highly selective ROCK2 kinase inhibitor, KD025, in MDA-MB-231 cells. We found that KD025 treatment decreased the phosphorylation of residues in the C-terminal (Ser423/425) and linker region (Ser204 and Ser208) of SMAD3, as well as in the SMAD2 C-terminal (Ser465/467), after TGF-β stimulation for 6 h (Fig. 3A). We also found that treatment with KD025 significantly reduced TGF-β-induced CAGA_12_-luciferase expression in MDA-MB-231 cells, whereas treatment with GSK429286A induced a slight increase (Fig. 3B). Treatment with KD025 also significantly decreased the mRNA levels of TGF-β target genes, such as *SERPINE1* and *SMAD7* in MDA-MB-231 cells (Fig. 4A and B), in NMuMG cells (Fig. 4C and D), in MCF 10 A MII cells (Fig. 4E and F), whereas treatment with GSK429286A had less or no effect. In A549 cells, expressing high level of ROCK2 (see below, Supplementary Figure S6C and D), treatment with KD025 inhibited *SERPINE1* expression (Supplementary Figure S3C), whereas this was not the case in BT-549 cells, expressing low level of ROCK2 (Supplementary Figure S3D and Supplementary S6C and D). These findings were further validated by depletion of individual ROCK isoforms using siRNA in MDA-MB-231 cells treated with TGF-ꞵ for 1–6 h, which revealed that ROCK2 knockdown dramatically reduced the C-terminal phosphorylation levels of SMAD2 and SMAD3 and also phosphorylation in the SMAD3 linker region, while ROCK1 knockdown enhanced SMAD3 C-terminal phosphorylation (Fig. 3C and D). Similarly, KD025 treatment of NMuMG cells decreased the phosphorylation of SMAD3 in the C-terminal (Ser423/425) and linker region (Ser204 and Ser208), as well as phosphorylation of SMAD2 in the C-terminal (Ser465/467) and linker region (Ser245, Ser250 and Ser255), after TGF-β stimulation for 24 h (Supplementary Figure S3A). In addition, treatment with K025 significantly reduced CAGA_12_-luciferase expression in NMuMG cells (Supplementary Figure S3B). The knockdown efficiencies of ROCK isoform mRNAs by the different siRNA and shRNA, as well as their effects on the mRNA expression of *SERPINE1*, are shown in Supplementary Figure S4; similarly, effects on ROCK isoform protein levels are shown in Fig. 3C and D.

Taken together, these findings show that inhibition of the ROCK2 kinase or knockdown of ROCK2 inhibits TGF-β-SMAD signaling, whereas ROCK1 has the opposite effect.

### ROCK2 inhibition prevents SMAD3 and SMAD4 nuclear translocation

**Fig. 5:**
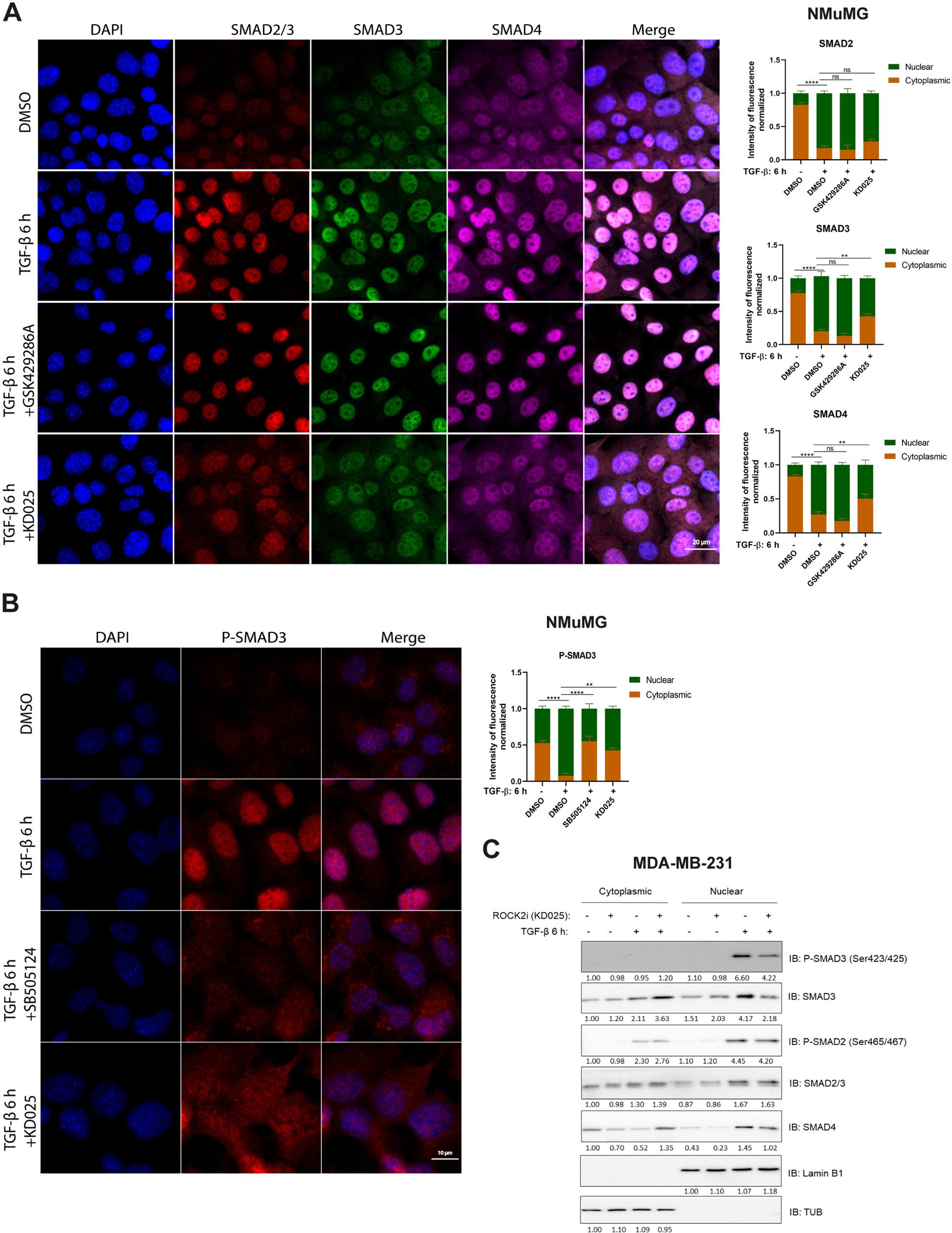
ROCK2 inhibition prevents SMAD3 and SMAD4 nuclear localization. **A**, **B** NMuMG cells were starved in medium, supplemented with 0.2% FBS, treated or not with GSK429286A (10 μM), KD025 (5 μM), SB505124 (2.5 µM) and TGF-β (5 ng/ml) for 6 h, and then subjected to immunofluorescence staining of antibodies against SMAD2/3, SMAD3 and SMAD4 (**A**; scale bar indicates 20 µm), or an antibody against Phospho-SMAD3 (**B**; scale bar indicates 10 µm); nuclei were visualized by DAPI staining. The intensity of fluorescence in cytoplasmic and nuclear fractions was quantified by Fiji (Image J) software. Data are presented in right panels **A** and **B** as mean ± SEM from three biological replicates; statistical significance was assessed by two-way ANOVA followed by Dunnett’s multiple comparisons test. ns, not significant difference; **, *p*< 0.01; ****, *p*< 0.0001. **C** Cytoplasmic and nuclear fractionations of MDA-MB-231 cells, treated or not with TGF-β (5 ng/ml) and KD025 (5 μM), were prepared and subjected to IB with the indicated antibodies. The band intensities were quantified and normalized to the cytoplasmic control condition set 1.00, and further normalized to Tubulin (TUB) or Lamin B1, by Image Lab software

To further explore the mechanism by which ROCK isoforms affect TGF-ꞵ-SMAD signaling, fluorescence immunostaining of NMuMG cells was performed by using antibodies against SMAD2/3, SMAD3 and SMAD4, as well as an antibody against the C-terminal phosphorylation (p) of SMAD3. As shown in Fig. 5A, TGF-β treatment for 6 h enhanced SMAD2, SMAD3 and SMAD4 nuclear accumulation, resulting in a low cytoplasmic, but high nuclear level of activated SMAD complex (see the merged images). ROCK2 inhibition by KD025 prevented the nuclear accumulation of SMAD3 and SMAD4, and to a lesser extent of SMAD2 (see quantification of fluorescence in Fig. 5A), while there was no significant effect after treatment with the pan-ROCK inhibitor (GSK429286A). Similar results of SMAD3 subcellular distribution were also found after treatment of MDA-MB-231 cells with the same ROCK inhibitors after stimulation with TGF-ꞵ for 24 h (Supplementary Figure S5A). Immunostaining with an antibody against C-terminal p-SMAD3 also revealed its decreased nuclear accumulation after treatment with KD025 (Fig. 5B). These findings were further validated by subcellular fractionation assay in MDA-MB-231 cells, ROCK2 inhibition by KD025 reduced the nuclear accumulation of p-SMAD3, SMAD3 and SMAD4 (Fig. 5C). Consistent with pharmacological inhibition by KD025, subcellular fractionation assays also revealed that siRNA-mediated depletion of ROCK2 decreased SMAD3 and SMAD4 protein levels in the nuclear fraction, while knockdown of ROCK1 had minimal effects on SMAD3 and SMAD4 nuclear localization (Supplementary Figure S5B and C).

### SMAD3 is selectively phosphorylated by ROCK2

To determine whether ROCK1 or ROCK2 directly phosphorylates SMAD3, we performed in vitro kinase assays using recombinant ROCK1 or ROCK2, and SMAD3 proteins, with kinase activities quantified by the ADP-Glo™ Kinase Assay kit, which measures ATP consumption through ADP production. As shown in Supplementary Figure S3E and F, SMAD3 was significantly phosphorylated by ROCK2, but not by ROCK1.

### SMAD3 binds ROCK1, but not ROCK2

**Fig. 6:**
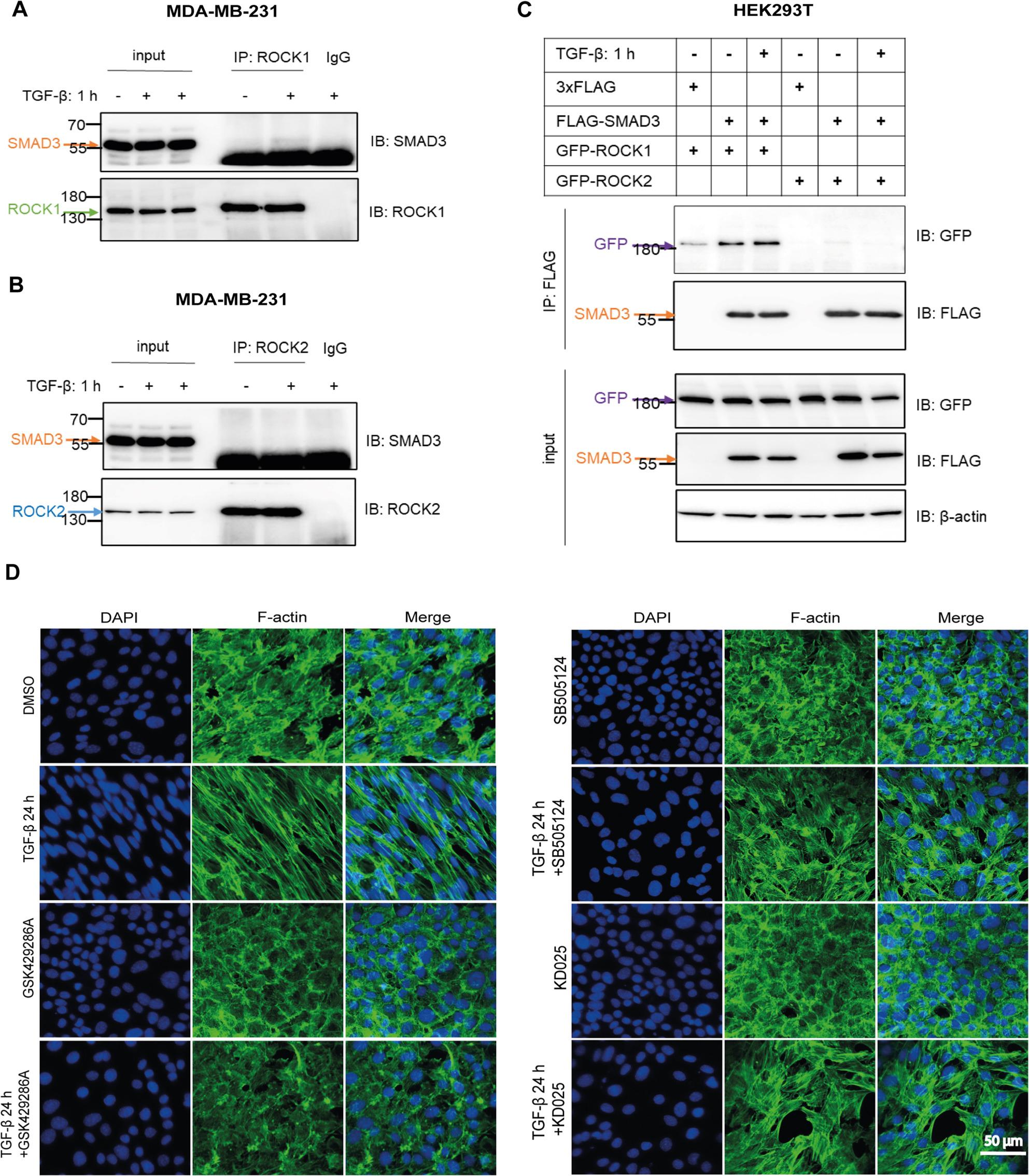
SMAD3 binds ROCK1, but not ROCK2, and ROCK kinase inhibition affects TGF-ꞵ-induced actin cytoskeleton reorganization. **A**, **B** Co-immunoprecipitation of endogenous SMAD3 and ROCK1 or ROCK2. MDA-MB-231 cells were seeded, starved and then stimulated or not with TGF-β (5 ng/ml) for 60 min. Cell lysates were then subjected to IP with a SMAD3 antibody, and IB with the indicated ROCK1 (**A**) or ROCK2 (**B**) antibodies. **C** Co-immunoprecipitation of SMAD3 and ROCK1 or ROCK2 after overexpression by transfection. HEK293T cells were transfected with 3×FLAG, FLAG-SMAD3, GFP-ROCK1 and GFP-ROCK2 plasmids; cell lysates were then subjected to IP with a FLAG antibody, followed by IB with the indicated antibodies. **D** Phalloidin staining of NMuMG cells treated or not with SB505124 (2.5 μM), GSK429286A (10 μM), KD025 (5 μM), and TGF-β (5 ng/ml) for 24 h. Scale bar, 50 µm

To study whether ROCK1 and ROCK2 interact with SMAD proteins, we performed co-immunoprecipitation experiments in MDA-MB-231 cells. We found that, at the endogenous level, SMAD3 bound ROCK1 in a TGF-β-dependent manner, whereas no binding of ROCK2 was observed (Fig. 6A and B). Moreover, an interaction between SMAD3 and ROCK1, but not with ROCK2, was further validated by co-immunoprecipitation experiment using HEK293T cells transfected with SMAD3, ROCK1 and ROCK2 plasmids (Fig. 6C).

### ROCK1 is mainly localized in the cytoplasm, whereas ROCK2 is mainly localized in the nucleus

To determine the subcellular localization of ROCK1 and ROCK2, lysates from MDA-MB-231 cells (Supplementary Figure S6A) and NMuMG cells (Supplementary Figure S6B), treated or not with TGF-β and the ROCK inhibitor GSK429286A, were prepared and subjected to subcelluar fractionation. ROCK1 was found to be mainly localized in the cytoplasm, whereas ROCK2 was mainly localized in the nucleus in both cell lines. Moreover, the cellular distributions of ROCK isoforms were not changed by GSK429286A treatment (Supplementary Figure S6A and B).

We also found that mRNA and protein expression levels of ROCK isoforms varied across cell lines of distinct biological origin. ROCK1 expression was high in human triple-negative breast cancer (TNBC) (MDA-MB-231, HCC38, BT-549), HER2-positive breast cancer (HCC1954 and MDA-MB-453), HER2-negative breast cancer (MCF-7), as well as in human embryonic kidney (HEK293T) and human keratinocyte (HaCaT) cell lines. ROCK2 expression was prominent in TNBC cell lines (MDA-MB-231 and Hs578T), Ras-transformed human mammary epithelial (MCF10A MII), human non-small cell lung cancer (NSCLC) (A549), and human osteosarcoma (U2OS) cell lines (Supplementary Figure S6C and D). Since the two ROCK isoforms have opposite effects on TGF-ꞵ signaling, this finding suggests that the effects of activation of the Rho-ROCK pathway on TGF-ꞵ signaling varies among different cell types.

### ROCK1 Inhibition disrupts the actin cytoskeleton

To determine the effects of ROCK isoforms on the organization of the actin cytoskeleton filaments of cells, NMuMG cells were treated with kinase inhibitors of ROCK isoforms and TβRI, for comparison. As shown in Fig. 6D, phalloidin staining of NMuMG cells revealed that TGF-β treatment for 24 h induced actin re-arrangement, which was reversed by the TꞵRI inhibitor SB505124 or by the pan-ROCK kinase inhibitor GSK429286A, however, the stress fiber assembly was not changed to the same extent after treatment with the highly selective ROCK2 kinase inhibitor KD025. This suggests that ROCK1 plays a more important role in regulating actin dynamics, than ROCK2.

### ROCK1 and ROCK2 have opposing effects on cell proliferation and invasion

**Fig. 7:**
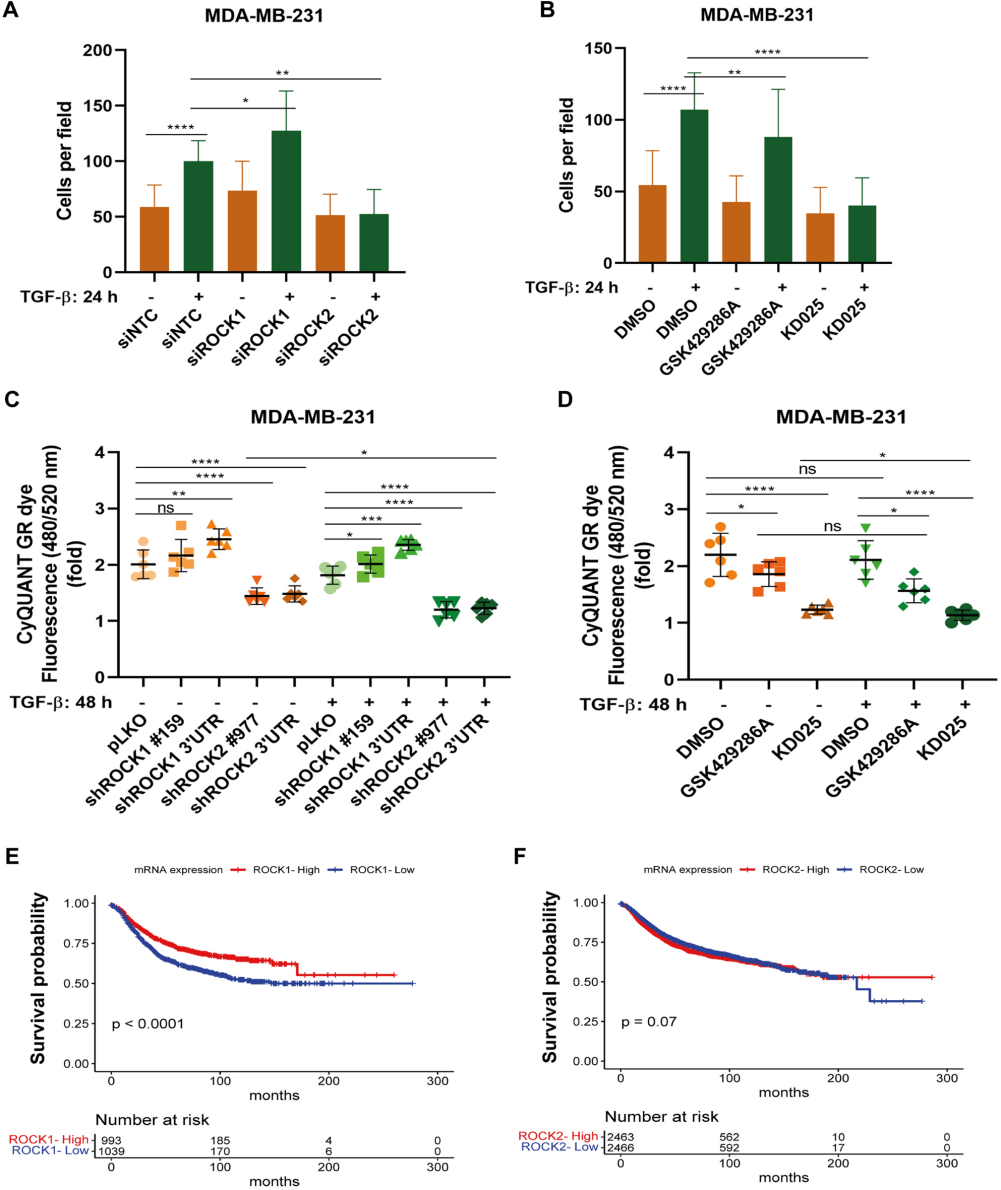
ROCK1 and ROCK2 have opposing effects on cell proliferation and invasion and their expression differently correlates with relapse-free survival of breast cancer patients. **A**, **B** MDA-MB-231 cells transfected with siRNA for negative control, ROCK1 or ROCK2 (**A**), or treated or not with GSK429286A (10 μM) or KD025 (5 μM) for 30 min (**B**), were stimulated with TGF-β (5 ng/ml) for 24 ^4^h. Then cells (5× 10^4^) were seeded into the inserts of transwell plates, washed with PBS after 16 h, fixed with ice-cold methanol and incubated with DAPI. Cells were thereafter counted and images were captured by a 90i time-lapse microscope. **C**, **D** MDA-MB-231-pLKO, shROCK1 and shROCK2 stable cells were seeded in 48-well plates (5000 cells/well), serum-starved overnight, and then treated or not with TGF-β (5 ng/ml), as indicated (**C**). MDA-MB-231 cells were treated or not with TGF-β (5 ng/ml), in the absence or presence of GSK429286A (10 μM) or KD025 (5 μM) (**D**). The fluorescence signal was measured by CyQUANT® GR Cell proliferation assay kit after 48 h. Data are presented in panels A-D as mean ± SD of at least three independent experiments; statistical significance was assessed by two-tailed unpaired Student’s t-tests. ns, not significant difference; *, *p*<0.05; **, *p*< 0.01; ***, *p*< 0.001; ****, *p*< 0.0001. **E**, **F** Kaplan-Meier analysis of correlations between ROCK1 (**E**) and ROCK2 (**F**) mRNA expression and relapse-free survival (RFS) of patients; data taken from the TCGA database by log-rank test. The number of patients at risk is indicated at the bottom during four times periods and *p*-values indicate statistically significant (or not) difference across the timeline

To explore whether ROCK1 or ROCK2 could regulate cell invasion in *vitro*, we performed a transwell cell invasion assay using MDA-MB-231 cells. As shown in Fig. 7A, ROCK1 knockdown significantly increased cell invasion, whereas ROCK2 knockdown significantly decreased TGF-ꞵ-induced cell invasion. The highly selective ROCK2 kinase inhibitor KD025 significantly reduced TGF-ꞵ-induced cell invasion, and the pan-ROCK inhibitor GSK429286A also suppressed MDA-MB-231 cells invasion, albeit less efficiently (Fig. 7B).

We also investigated whether knockdown of ROCK1 or ROCK2 influenced MDA-MB-231 cell proliferation. As shown in Fig. 7C, ROCK2 knockdown significantly deceased cell proliferation in *vitro*, whereas ROCK1 knockdown increased cell proliferation, with or without TGF-β treatment. Moreover, treatment with the ROCK2 kinase inhibitor KD025 suppressed cell proliferation, with or without TGF-ꞵ stimulation; the inhibition of both ROCK isoforms by GSK429286A also suppressed cell proliferation, but less efficiently (Fig. 7D).

### The expression of ROCK1 and ROCK2 differently correlate with prognosis of breast cancer patients

Analyses of patients with breast cancer in the TCGA database using the Kaplan-Meier Plotter dataset, revealed that high *ROCK1* mRNA level significantly correlated with prolonged relapse-free survival (RFS) time of patients compared to patients with low mRNA level of *ROCK1* (Fig. 7E). In contrast, there was no significant correlation between *ROCK2* mRNA expression and RFS of patients with breast cancer (Fig. 7F).

**Fig. 8:**
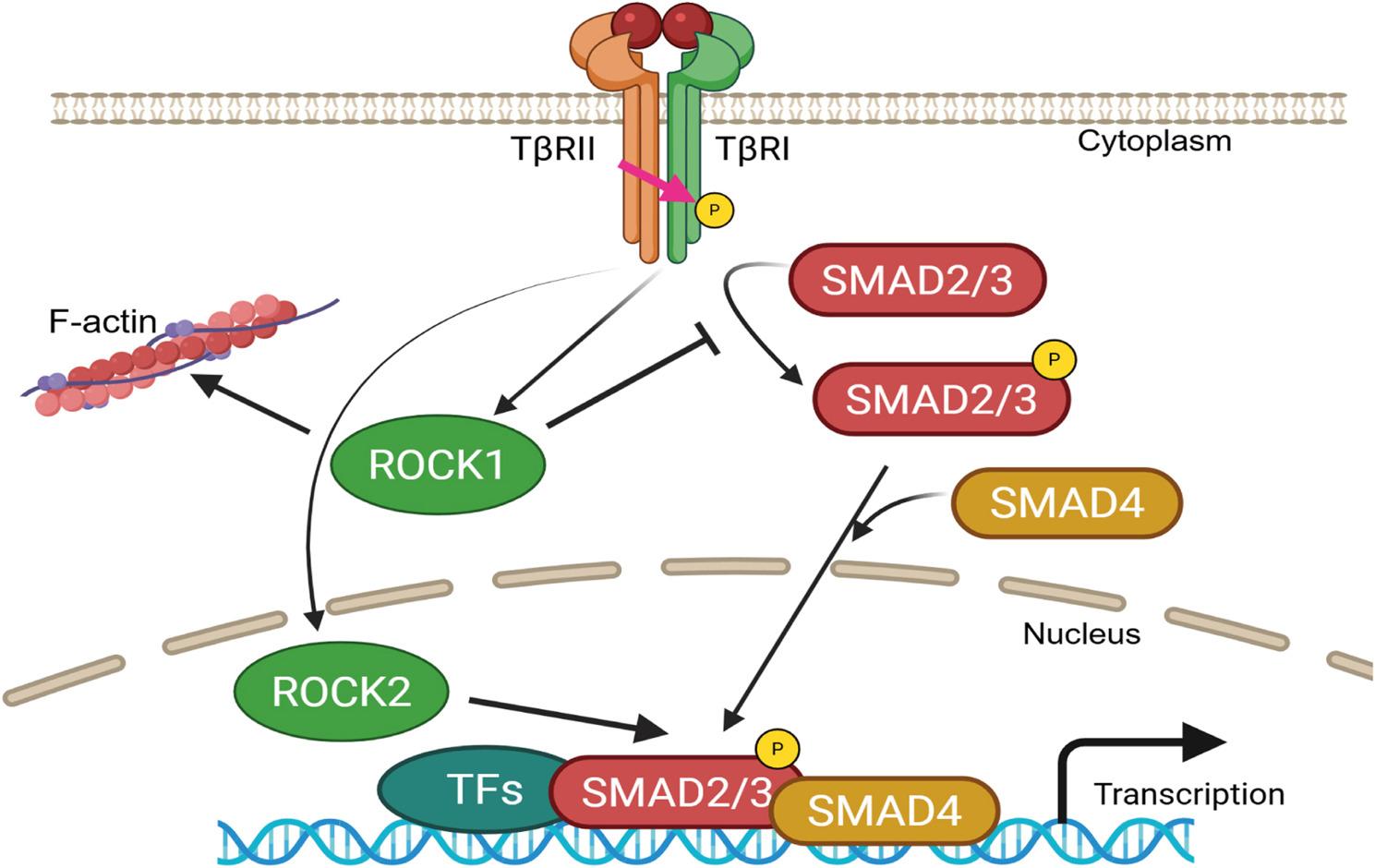
Schematic illustration of the opposite effects of ROCK1 and ROCK2 in regulation of TGF-ꞵ-SMAD signaling. ROCK1, localized predominantly in the cytoplasm, interacts with TβRI and SMAD3 to inhibit transcriptional activity. In contrast, ROCK2, primarily localized in the nucleus, facilitates SMAD3/4 nuclear accumulation and promotes the expression of TGF-β-SMAD target genes. (Created in Biorender)

Taken together, our observations support the notion that ROCK1 inhibits, whereas ROCK2 enhances, TGF-β-SMAD signaling (Fig. 8).

## Discussion

In the present study, we demonstrate that knockdown of ROCK1 and ROCK2 suppresses and enhances TGF-β-SMAD signaling, respectively. In contrast, the overexpression of ROCK1 and ROCK2 inhibits and promotes TGF-β-induced CAGA_12_-luc reporter activities, respectively. Thus, the two ROCK isoforms exert opposite effects in TGF-ꞵ signaling. In addition, expression of a kinase-dead ROCK1 mutant or treatment with a pan-ROCK kinase inhibitor reversed the ROCK1 effect, whereas expression of a ROCK2 kinase-dead mutant or treatment with a ROCK2-specific kinase inhibitor reversed the effect of ROCK2, suggesting that the kinase activities of ROCK1 and ROCK2 are needed to exert their effects on TGF-β signaling. Furthermore, we found that ROCK2 inhibition by knockdown of ROCK2 or treatment with a ROCK2 specific kinase inhibitor (KD025) reduced SMAD3 C-terminal (Ser423/425) and linker region (Ser204 and Ser208) phosphorylation. Moreover, the expression of the TGF-ꞵ target genes *SERPINE1* and *SMAD7* was reduced in MDA-MB-231 cells, NMuMG cells and MCF10A MII cells. Moreover, ROCK1 knockdown enhanced SMAD3 C-terminal (Ser423/425) phosphorylation and *SERPINE1* expression in MDA-MB-231 cells. We elucidated a possible mechanism for the different effects of the ROCK isoforms, by showing that pharmacological ROCK2 inhibition or siRNA-mediated depletion of ROCK2 prevents the nuclear accumulation of SMAD3 and SMAD4, but has no effect on SMAD2 nuclear accumulation; In contrast, depletion of ROCK1 has less or no effect, indicating ROCK isoform-dependent regulation of TGF-ꞵ-SMAD signaling. It remains to be determined whether the effect on nuclear accumulation is due to enhanced nuclear translocation, suppressed nuclear export, or effects on the phosphorylation status of SMAD3 and SMAD4.

It is well-known that TGF-β activates the Rho-ROCK signaling pathway [5, 30]. For example, TGF-β was shown to activate rapidly a Rho/ROCK/LIMK2/cofilin pathway regulating early actin cytoskeleton reorganization [31]. TGF-β treatment activated RhoA in epithelial cells in less than 15 min, and blocking of RhoA or its downstream target ROCK1 by the expression of dominant-negative mutants, led to inhibition of TGF-β mediated induction of actin stress fibers in NMuMG cells [32]. In PC-3U human prostate carcinoma cells, TGF-β stimulation resulted in the activation of CDC42 and RhoA, with a peak at 12 h. Long-term stimulation with TGF-β (48 h) induced the assembly of stress fibers, which required the activity of Rho GTPases, CDC42, p38-MAP Kinase, RhoA-ROCK and SMAD4 [33]. However, the mechanisms by which ROCK isoforms affect TGF-β-SMAD signaling remained unclear.

We found that ROCK1 and ROCK2 showed opposing effects on MDA-MB-231 cell proliferation and invasion. We observed a significant increase in cell proliferation and invasion in ROCK1-deficient cells, but a decrease in ROCK2-deficient cells, relative to the control cells. Moreover, ROCK2 knockdown or treatment with ROCK inhibitors suppressed the proliferation and invasion of the human breast cancer cell line MDA-MB-231 in *vitro*. It has been shown that inhibition of ROCK activity reduces proliferation, migration and invasion of certain types of cells, such as glioblastoma cells [34], melanoma cells [35], human bladder cancer cells [36], ovarian cancer cells [37], murine gastric cancer cells [38], and human breast cancer cells [39–43]. On the contrary, ROCK inhibition was found to increase cell proliferation, migration and invasion of SW620 human colon cancer cells [44], to promote the acquisition of a migratory phenotype and invasive capacities [45], and to increase the migration of SW480 human colon cancer cells with significantly altered focal adhesions [46]. Treatment with the pan-ROCK inhibitor Y-27,632 resulted in inhibition of epidermal keratinocyte differentiation and an increase in cell proliferation [47].

Moreover, previous studies have reported a cross-talk between Rho-ROCK and the TGF-β-SMAD pathway, and the ability of ROCK to inhibit or enhance phosphorylation of SMAD2/3 [48, 49]. For instance, ROCK inhibition by Y-27,632 promoted TGF-β3-induced tenogenic differentiation of mesenchymal stromal cells (MSC), with increased SMAD2/3 nuclear translocation, thereby activating canonical TGF-β3 signaling [50]. Y-27,632 treatment also reduced SMAD2 linker phosphorylation at Ser245/250/255, but only in MSCs cultured on collagen matrix, while Y-27,632 increased SMAD2 linker phosphorylation at Ser245/250/255 and SMAD3 linker phosphorylation at Ser204 in monolayer cultures [51]. However, another study reported that ROCK inhibition by Y-27,632 decreased SMAD3 linker phosphorylation at Ser204 in the human breast cancer cell line MCF10CA1h. Moreover, a p38 MAP-kinase inhibitor (SB203580) and Y-27,632 downregulated the linker region phosphorylation of SMAD3 at Ser203 and Ser207, and altered the phosphorylation of SMAD2/3 at Thr8, Thr178 and Ser212 [52]. Furthermore, Y-27,632 significantly decreased TGF-β induced SMAD3 phosphorylation at the C-terminal (Ser423/425) and also at the linker region (Ser203), while Y-27,632 had weak effects on SMAD2 phosphorylation at the linker region (Ser245/250/255) in ocular Tenon’s capsule fibroblasts (OTFs) cells [53].

Together, these studies reveal that inhibition of ROCK isoforms has different effects on cancer cell proliferation, migration and invasion. One explanation for these differences may be related to our finding that ROCK1 and ROCK2 exert opposite effects on TGF-β signaling. Thus, the relative expression levels of the two ROCK isoforms may determine the effect of inhibition of the two ROCK isoforms. This hypothesis is further supported by our analysis of the protein and mRNA expressions of the two ROCK isoforms in different cell lines (Supplementary Figure S6), as well as the gene expression analysis in the report of [54], which revealed that *ROCK1* mRNA was highly expressed in nine breast cancer cells (MDA-MB-231, -463, -453, -436, -468, -474, ZR-75-1, MCF-7, BT-482), while *ROCK2* expression was high in MDA-MB-231 and -436 cells compared to normal mammary epithelial cells.

We found that the TβRI inhibitor SB505124 and the pan-ROCK inhibitor GSK429286A reversed TGF-β-induced stress fiber formation, whereas treatment with the highly selective ROCK2 inhibitor KD025 had a less pronounced effect on actin organization, as visualized by phalloidin staining of NMuMG cells. These findings are consistent with previous studies that demonstrated a pivotal role of ROCK1 in the formation of stress fibers, whereas ROCK2-mediated cortical contractility and phagocytosis of fibronectin in fibroblasts, both of which were dependent on myosin light chain (MLC) phosphorylation [55, 56].

In our study, the immunoprecipitation assay followed by mass spectrometric analysis revealed that TβRI interacts with ROCK1, and that the interaction was enhanced after TGF-β stimulation for 60 min. We further confirmed an interaction of TβRI and ROCK1, but not with ROCK2, by co-immunoprecipitation. Furthermore, we found that SMAD3 interacts with ROCK1, but not with ROCK2, which may be related to the inhibitory effect of ROCK1 on TGF-ꞵ-SMAD signaling. In contrast, in vitro kinase assays demonstrated that SMAD3 is phosphorylated by ROCK2, but not by ROCK1, indicating that ROCK2 directly targets SMAD3 through its kinase activity.

In conclusion, our findings support a model in which ROCK1 and ROCK2 exert opposing and kinase-dependent effects on TGF-β-SMAD signaling. ROCK1 acts as a negative regulator, interacting with TβRI and SMAD3 to restrain transcriptional activity, while ROCK2 functions as a positive regulator, likely by enhancing SMAD3/4 nuclear accumulation promoting TGF-ꞵ-SMAD-dependent gene expression. Together, these findings establish a dual regulatory mechanism in which ROCK1 restrains, while ROCK2 enhances, TGF-β-SMAD-mediated transcription and cancer cell behaviour.

## Supplementary Information

Additional file 1. Table S1. Antibodies used for immunoblotting (IB) and immunofluorescence (IF). Table S2. Primer sequences used for the kinase-dead mutants of ROCK1 and ROCK2. Table S3. Primer sequences used for RT-qPCR.

Additional file 2. Table S4. Mass spectrometry data.

Additional file 3. Additional figures and legends.

Additional file 4. Uncropped immunoblots

## Data Availability

The mass spectrometry data are presented in Additional file 2 in Supplementary materials. Further details or additional information are available upon reasonable request.

## Abbreviations

CDC42: Cell division cycle 42
MAPK: Mitogen-activated protein kinase
ROCK: Rho-associated coiled-coil kinase
TGF-β: Transforming growth factor-β
TβRI: TGF-β type I receptor
TβRII: TGF-β type II receptor

## References

[1] Hao Y, Baker D, Ten Dijke P. TGF-beta-mediated epithelial- mesenchymal transition and cancer metastasis. Int J Mol Sci. 2019;20(11):2767. 10.3390/ijms20112767 PMC6600375 31195692

[2] Heldin CH, Moustakas A. Signaling receptors for TGF-beta family members. Cold Spring Harb Perspect Biol. 2016;8(8):a022053. 10.1101/cshperspect.a022053 PMC4968163 27481709

[3] Massague J, Blain SW, Lo RS. TGFbeta signaling in growth control, cancer, and heritable disorders. Cell. 2000;103(2):295–309. 11057902 10.1016/s0092-8674(00)00121-5

[4] Matsuura I, et al. Cyclin-dependent kinases regulate the antiproliferative function of Smads. Nature. 2004;430(6996):226–31. 15241418 10.1038/nature02650

[5] Ma J, et al. TGF-beta-Induced endothelial to mesenchymal transition in disease and tissue engineering. Front Cell Dev Biol. 2020;8:260. 32373613 10.3389/fcell.2020.00260 PMC7187792

[6] Yakymovych I, et al. The type II TGF-beta receptor phosphorylates Tyr(182) in the type I receptor to activate downstream Src signaling. Sci Signal. 2022;15(760):eabp9521. 36378749 10.1126/scisignal.abp9521

[7] Rath N, Olson MF. Rho-associated kinases in tumorigenesis: re-considering ROCK Inhibition for cancer therapy. EMBO Rep. 2012;13(10):900–8. 22964758 10.1038/embor.2012.127 PMC3463970

[8] Matsuoka T, Yashiro M. Rho/ROCK signaling in motility and metastasis of gastric cancer. World J Gastroenterol. 2014;20(38):13756–66. 25320513 10.3748/wjg.v20.i38.13756 PMC4194559

[9] Wei L, et al. Novel insights into the roles of Rho kinase in cancer. Arch Immunol Ther Exp (Warsz). 2016;64(4):259–78. 26725045 10.1007/s00005-015-0382-6 PMC4930737

[10] Wen W, et al. Structure basis and unconventional lipid membrane binding properties of the PH-C1 tandem of Rho kinases. J Biol Chem. 2008;283(38):26263–73. 18640982 10.1074/jbc.M803417200 PMC3258851

[11] Amano M, Nakayama M, Kaibuchi K. Rho-kinase/ROCK: A key regulator of the cytoskeleton and cell Polarity. Cytoskeleton (Hoboken). 2010;67(9):545–54. 20803696 10.1002/cm.20472 PMC3038199

[12] Julian L, Olson MF. Rho-associated coiled-coil containing kinases (ROCK): structure, regulation, and functions. Small GTPases. 2014;5:e29846. 25010901 10.4161/sgtp.29846 PMC4114931

[13] Shi J, Wei L. Rho kinases in embryonic development and stem cell research. Arch Immunol Ther Exp (Warsz). 2022;70(1):4. 35043239 10.1007/s00005-022-00642-z PMC8766376

[14] Street CA, Bryan BA. Rho kinase proteins–pleiotropic modulators of cell survival and apoptosis. Anticancer Res. 2011;31(11):3645–57. 22110183 PMC3226732

[15] Tsai PC, et al. Taiwan Cobra cardiotoxin III suppresses EGF/EGFR-mediated epithelial-to-mesenchymal transition and invasion of human breast cancer MDA-MB-231 cells. Toxicon. 2016;111:108–20. 26774845 10.1016/j.toxicon.2016.01.051

[16] Kumar P, Aggarwal R. An overview of triple-negative breast cancer. Arch Gynecol Obstet. 2016;293(2):247–69. 26341644 10.1007/s00404-015-3859-y

[17] Nagumo H, et al. Rho kinase inhibitor HA-1077 prevents Rho-mediated myosin phosphatase Inhibition in smooth muscle cells. Am J Physiol Cell Physiol. 2000;278(1):C57–65. 10644512 10.1152/ajpcell.2000.278.1.C57

[18] Uehata M, et al. Calcium sensitization of smooth muscle mediated by a Rho-associated protein kinase in hypertension. Nature. 1997;389(6654):990–4. 9353125 10.1038/40187

[19] Goodman KB, et al. Development of dihydropyridone Indazole amides as selective Rho-kinase inhibitors. J Med Chem. 2007;50(1):6–9. 17201405 10.1021/jm0609014

[20] Nichols RJ, et al. Substrate specificity and inhibitors of LRRK2, a protein kinase mutated in parkinson’s disease. Biochem J. 2009;424(1):47–60. 19740074 10.1042/BJ20091035 PMC3759966

[21] Boerma M, et al. Comparative gene expression profiling in three primary human cell lines after treatment with a novel inhibitor of Rho kinase or Atorvastatin. Blood Coagul Fibrinolysis. 2008;19(7):709–18. 18832915 10.1097/MBC.0b013e32830b2891 PMC2713681

[22] Barcelo J, Samain R, Sanz-Moreno V. Preclinical to clinical utility of ROCK inhibitors in cancer. Trends Cancer. 2023;9(3):250–63. 36599733 10.1016/j.trecan.2022.12.001

[23] Lee MH, et al. Targeting ROCK/LIMK/cofilin signaling pathway in cancer. Arch Pharm Res. 2019;42(6):481–91. 31030376 10.1007/s12272-019-01153-w

[24] Priya R, et al. Feedback regulation through myosin II confers robustness on RhoA signalling at E-cadherin junctions. Nat Cell Biol. 2015;17(10):1282–93. 26368311 10.1038/ncb3239

[25] Truebestein L, et al. A molecular ruler regulates cytoskeletal remodelling by the Rho kinases. Nat Commun. 2015;6:10029. 26620183 10.1038/ncomms10029 PMC4686654

[26] Moggridge S, et al. Extending the compatibility of the SP3 paramagnetic bead processing approach for proteomics. J Proteome Res. 2018;17(4):1730–40. 29565595 10.1021/acs.jproteome.7b00913

[27] Tang D, et al. SRplot: A free online platform for data visualization and graphing. PLoS ONE. 2023;18(11):e0294236. 37943830 10.1371/journal.pone.0294236 PMC10635526

[28] Ge SX, Jung D, Yao R. ShinyGO: a graphical gene-set enrichment tool for animals and plants. Bioinformatics. 2020;36(8):2628–9. 31882993 10.1093/bioinformatics/btz931 PMC7178415

[29] Dennler S, et al. Direct binding of Smad3 and Smad4 to critical TGF beta-inducible elements in the promoter of human plasminogen activator inhibitor-type 1 gene. EMBO J. 1998;17(11):3091–100. 9606191 10.1093/emboj/17.11.3091 PMC1170648

[30] Kardassis D, et al. Control of transforming growth factor beta signal transduction by small GTPases. FEBS J. 2009;276(11):2947–65. 19490100 10.1111/j.1742-4658.2009.07031.x

[31] Vardouli L, Moustakas A, Stournaras C. LIM-kinase 2 and Cofilin phosphorylation mediate actin cytoskeleton reorganization induced by transforming growth factor-beta. J Biol Chem. 2005;280(12):11448–57. 15647284 10.1074/jbc.M402651200

[32] Bhowmick NA, et al. Integrin beta 1 signaling is necessary for transforming growth factor-beta activation of p38MAPK and epithelial plasticity. J Biol Chem. 2001;276(50):46707–13. 11590169 10.1074/jbc.M106176200

[33] Edlund S, et al. Transforming growth factor-beta-induced mobilization of actin cytoskeleton requires signaling by small GTPases Cdc42 and RhoA. Mol Biol Cell. 2002;13(3):902–14. 11907271 10.1091/mbc.01-08-0398 PMC99608

[34] Zohrabian VM, et al. Rho/ROCK and MAPK signaling pathways are involved in glioblastoma cell migration and proliferation. Anticancer Res. 2009;29(1):119–23. 19331140

[35] Routhier A, et al. Pharmacological Inhibition of Rho-kinase signaling with Y-27632 blocks melanoma tumor growth. Oncol Rep. 2010;23(3):861–7. 20127030

[36] Abe H, et al. The Rho-kinase inhibitor HA-1077 suppresses proliferation/migration and induces apoptosis of urothelial cancer cells. BMC Cancer. 2014;14:412. 24908363 10.1186/1471-2407-14-412 PMC4081468

[37] Li Y, et al. Visfatin derived from Ascites promotes ovarian cancer cell migration through Rho/ROCK signaling-mediated actin polymerization. Eur J Cancer Prev. 2015;24(3):231–9. 25055182 10.1097/CEJ.0000000000000064

[38] Hinsenkamp I, et al. Inhibition of Rho-Associated kinase 1/2 attenuates tumor growth in murine gastric cancer. Neoplasia. 2016;18(8):500–11. 27566106 10.1016/j.neo.2016.07.002 PMC5018096

[39] Guerra FS, et al. ROCK Inhibition with fasudil induces beta-catenin nuclear translocation and inhibits cell migration of MDA-MB 231 human breast cancer cells. Sci Rep. 2017;7(1):13723. 29057980 10.1038/s41598-017-14216-z PMC5651822

[40] Leonel C, et al. Inhibition of Epithelial-mesenchymal transition in response to treatment with Metformin and Y27632 in breast cancer cell lines. Anticancer Agents Med Chem. 2017;17(8):1113–25. 28042775 10.2174/1871520617666170102153954

[41] Liu S, et al. Inhibition of rho-associated kinase signaling prevents breast cancer metastasis to human bone. Cancer Res. 2009;69(22):8742–51. 19887617 10.1158/0008-5472.CAN-09-1541

[42] Matsubara M, Bissell MJ. Inhibitors of Rho kinase (ROCK) signaling revert the malignant phenotype of breast cancer cells in 3D context. Oncotarget. 2016;7(22):31602–22. 27203208 10.18632/oncotarget.9395 PMC5077963

[43] Unbekandt M, et al. A novel small-molecule MRCK inhibitor blocks cancer cell invasion. Cell Commun Signal. 2014;12:54. 25288205 10.1186/s12964-014-0054-x PMC4195943

[44] Vishnubhotla R, et al. Treatment with Y-27632, a ROCK Inhibitor, increases the proinvasive nature of SW620 cells on 3D collagen type 1 matrix. Int J Cell Biol. 2012;2012:p259142. 10.1155/2012/259142 PMC3368365 22690219

[45] Poisson L, et al. Rock Inhibition promotes Na(V)1.5 sodium channel-dependent SW620 colon cancer cell invasiveness. Sci Rep. 2020;10(1):13350. 32770034 10.1038/s41598-020-70378-3 PMC7414216

[46] Adachi S, et al. Rho-kinase inhibitor upregulates migration by altering focal adhesion formation via the Akt pathway in colon cancer cells. Eur J Pharmacol. 2011;650(1):145–50. 20959118 10.1016/j.ejphar.2010.10.014

[47] McMullan R, et al. Keratinocyte differentiation is regulated by the Rho and ROCK signaling pathway. Curr Biol. 2003;13(24):2185–9. 14680635 10.1016/j.cub.2003.11.050

[48] Kamato D, Little PJ. Smad2 linker region phosphorylation is an autonomous cell signalling pathway: implications for multiple disease pathologies. Biomed Pharmacother. 2020;124:109854. 31981946 10.1016/j.biopha.2020.109854

[49] Wang Y, et al. DLC1-dependent parathyroid hormone-like hormone Inhibition suppresses breast cancer bone metastasis. J Clin Invest. 2014;124(4):1646–59. 24590291 10.1172/JCI71812 PMC3973085

[50] Melzer M, et al. Rho/ROCK Inhibition promotes TGF-beta3-Induced tenogenic differentiation in mesenchymal stromal cells. Stem Cells Int. 2021;2021:p8284690. 10.1155/2021/8284690 PMC8519677 34659420

[51] Melzer M, et al. Differential Smad2/3 linker phosphorylation is a crosstalk mechanism of Rho/ROCK and canonical TGF-beta3 signaling in tenogenic differentiation. Sci Rep. 2024;14(1):10393. 38710741 10.1038/s41598-024-60717-z PMC11074336

[52] Kamaraju AK, Roberts AB. Role of Rho/ROCK and p38 MAP kinase pathways in transforming growth factor-beta-mediated Smad-dependent growth Inhibition of human breast carcinoma cells in vivo. J Biol Chem. 2005;280(2):1024–36. 15520018 10.1074/jbc.M403960200

[53] Feng ZH, et al. Involvement of Rho-associated coiled-coil kinase signaling Inhibition in TGF-beta1/Smad2, 3 signal transduction in vitro. Int J Ophthalmol. 2017;10(12):1805–11. 29259896 10.18240/ijo.2017.12.03 PMC5733505

[54] Lane J, et al. The expression and prognostic value of ROCK I and ROCK II and their role in human breast cancer. Int J Oncol. 2008;33(3):585–93. 18695890

[55] Wang Y, et al. ROCK isoform regulation of myosin phosphatase and contractility in vascular smooth muscle cells. Circ Res. 2009;104(4):531–40. 19131646 10.1161/CIRCRESAHA.108.188524 PMC2649695

[56] Yoneda A, Multhaupt HA, Couchman JR. The Rho kinases I and II regulate different aspects of myosin II activity. J Cell Biol. 2005;170(3):443–53. 16043513 10.1083/jcb.200412043 PMC2171463

