# Supplementary material for: Opposing effects of Rho-associated coiled-coil kinase 1 (ROCK1) and ROCK2 in TGF-β-SMAD signaling
Source: Cell Commun Signal. 2026 Feb 7;24:137. doi: 10.1186/s12964-026-02722-5 (PMC12930806; doi:10.1186/s12964-026-02722-5)
Supplement: Supplementary file 1 — Additional file 1. Table S1. Antibodies used for immunoblotting (IB) and immunofluorescence (IF). Table S2. Primer sequences used for the kinase-dead mutants of ROCK1 and ROCK2. Table S3. Primer sequences used for RT-qPCR. [file 12964_2026_2722_MOESM1_ESM.docx]

**Opposing effects of Rho-associated coiled-coil kinase 1 (ROCK1) and ROCK2**

**in TGF-β-SMAD signaling**

Yu Bai^1,2^*, Mohamad Moustafa Ali^1^, Maarten van Dinther^3^, Peter ten Dijke^3^, Aristidis Moustakas^1^, Anders Sundqvist^1,4^ and Carl-Henrik Heldin^1^*

^1^Department of Medical Biochemistry and Microbiology, Science for Life Laboratory, Box 582, Biomedical Center, Uppsala University, SE-75123 Uppsala, Sweden

^2^Present address: Department of Immunology, Genetics and Pathology, Science for Life Laboratory, Uppsala University, SE-751 85 Uppsala, Sweden

^3^Department of Cell and Chemical Biology, Oncode Institute, Leiden University Medical Center, Leiden, The Netherlands

^4^Department of Pharmaceutical Biosciences, Uppsala University, Sweden

**Running title**: Opposing Roles of ROCK1 and ROCK2 in TGF-β Signaling

*Corresponding authors: Yu Bai, Department of Immunology, Genetics and Pathology, Science for Life Laboratory, Uppsala University, SE-751 85 Uppsala, Sweden. E-mail: yu.bai@igp.uu.se

Carl-Henrik Heldin, Department of Medical Biochemistry and Microbiology, Science for Life Laboratory, Box 582, Biomedical Center, Uppsala University, SE-75123 Uppsala, Sweden. E-mail: c-h.heldin@imbim.uu.se

**Conflict of interest**: The authors declare that they have no conflict of interest.

| Antigen | Species | Source | Catalog. | Dilution |
| --- | --- | --- | --- | --- |
| ROCK1 | Rabbit | Cell Signaling Technology | #4035 | 1:1000 |
| ROCK2 | Rabbit | Cell Signaling Technology | #9029 | 1:1000 |
| phospho-Ser465/467 SMAD2 | Rabbit | Homemade [1] |  | 1:1000 |
| Phospho-Ser245/250/255 SMAD2 | Rabbit | Cell Signaling Technology | #3104 | 1:1000 |
| Phospho-Ser423/425 SMAD3 | Rabbit | Cell Signaling Technology | #9520 | 1:1000 |
| Phospho-Ser204 SMAD3 | Rabbit | Homemade [2] |  | 0.15 μg/ml |
| Phospho-Ser208 SMAD3 | Rabbit | Homemade [2] |  | 0.15 μg/ml |
| SMAD3 | Rabbit | Cell Signaling Technology | #9523 | 1:1000 |
| SMAD3 | Rabbit | Abcam | ab28379 | 1:1000 |
| SMAD2/3 | Mouse | BD Transduction Laboratories | #610843 | 1:1000 |
| SMAD4 | Mouse | Santa Cruz Technology | sc-7966 | 1:500 |
| TβRI | Rabbit | Abcam | ab31013 | 1:1000 |
| TβRII | Rabbit | Cell Signaling Technology | #41896 | 1:1000 |
| PAI1 | Mouse | BD Bioscience | #612024 | 1:1000 |
| Phospho-p38 MAPK | Rabbit | Cell Signaling Technology | #9211 | 1:1000 |
| HA-tag | Rabbit | Santa Cruz Technology | sc-805 | 1:1000 |
| FLAG-tag | Mouse | Sigma-Aldrich | F3165 | 1:1000 |
| GFP-tag | Mouse | ThermoFisher | #MA5-15256 | 1:2000 |
| β-actin | Mouse | ThermoFisher | #MA1-140 | 1:1000 |
| Tubulin | Mouse | Sigma-Aldrich | T0198 | 1:1000 |
| Lamin B1 | Rabbit | Abcam | ab16048 | 1:1000 |

Supplementary Table S1. **Antibodies used in this study for immunoblotting (IB) and immunofluorescence (IF).**

Supplementary Table S2. **Sequences of primers used in the study for the constructions of mutants of ROCK isoforms.**

| ROCK1 (K105R) forward | TTGCTGAGCAGCCTCATAGCATAAACCTTCCTAGTG |
| --- | --- |
| ROCK1 (K105R) reverse | CACTAGGAAGGTTTATGCTATGAGGCTGCTCAGCAA |
| ROCK2 (K121R) forward | GGCATCACAGAAGGTTTATGCAATGAGGCTTCTTAGTAAATTTG |
| ROCK2 (K121R) reverse | CAAATTTACTAAGAAGCCTCATTCATAAACCTTCTGTGATGCC |

Supplementary Table S3. **Sequences of primers used in the study for qRT-PCR.**

| Gene | Forward sequence | Reverse sequence |
| --- | --- | --- |
| Species: human | | |
| *SERPINE1* | GTGGTCTGTGTCACCGTATC | GTAGTTGAATCCGAGCTGCC |
| *SMAD7* | TGTCCAGATGCTGTGCCTTCCT | CTCGTCTTCTCCTCCCAGTATG |
| *ROCK1* | GAAACAGTGTTCCATGCTAGACG | GCCGCTTATTTGATTCCTGCTCC |
| *ROCK2* | TGCGGTCACAACTCCAAGCCTT | CGTACAGGCAATGAAAGCCATCC |
| *GAPDH* | GGAGTCAACGGATTTGGTCGTA | GGCAACAATATCCACTTTACCA |
| *JUNB* | ACTCATACACAGCTACGGGATACG | GGCTCGGTTTCAGGAGTTTG |
| Species: mouse | | |
| *SERPINE1* | GGCAGATCCAAGATGCTATGG | TCATTCTTGTTCCACGGCC |
| *SMAD7* | CTGGTGTGCTGCAACCCCCATC | ATCTGGACAGCCTGCAGTTGGTT |
| *GAPDH* | TGTGTCCGTCGTGGATCTGA | CCTGCTTCACCACCTTCTTGA |

1. Persson, U., et al., *The L45 loop in type I receptors for TGF-beta family members is a critical determinant in specifying Smad isoform activation.* FEBS Lett, 1998. 434(1-2): p. 83-7.

2. Wang, G., et al., *Transforming growth factor-beta-inducible phosphorylation of Smad3.* J Biol Chem, 2009. 284(15): p. 9663-73.

**References**
