# Supplementary material for: Opposing effects of Rho-associated coiled-coil kinase 1 (ROCK1) and ROCK2 in TGF-β-SMAD signaling
Source: Cell Commun Signal. 2026 Feb 7;24:137. doi: 10.1186/s12964-026-02722-5 (PMC12930806; doi:10.1186/s12964-026-02722-5)
Supplement: Supplementary file 3 — Additional file 3. Additional figures and legends. [file 12964_2026_2722_MOESM3_ESM.docx]

**Opposing effects of Rho-associated coiled-coil kinase 1 (ROCK1) and ROCK2**

**in TGF-β-SMAD signaling**

Yu Bai^1,2^*, Mohamad Moustafa Ali^1^, Maarten van Dinther^3^, Peter ten Dijke^3^, Aristidis Moustakas^1^, Anders Sundqvist^1,4^ and Carl-Henrik Heldin^1^*

^1^Department of Medical Biochemistry and Microbiology, Science for Life Laboratory, Box 582, Biomedical Center, Uppsala University, SE-75123 Uppsala, Sweden

^2^Present address: Department of Immunology, Genetics and Pathology, Science for Life Laboratory, Uppsala University, SE-751 85 Uppsala, Sweden

^3^Department of Cell and Chemical Biology, Oncode Institute, Leiden University Medical Center, Leiden, The Netherlands

^4^Department of Pharmaceutical Biosciences, Uppsala University, Sweden

**Running title**: Opposing Roles of ROCK1 and ROCK2 in TGF-β Signaling

*Corresponding authors: Yu Bai, Department of Immunology, Genetics and Pathology, Science for Life Laboratory, Uppsala University, SE-751 85 Uppsala, Sweden. E-mail: yu.bai@igp.uu.se

Carl-Henrik Heldin, Department of Medical Biochemistry and Microbiology, Science for Life Laboratory, Box 582, Biomedical Center, Uppsala University, SE-75123 Uppsala, Sweden. E-mail: c-h.heldin@imbim.uu.se

**Conflict of interest**: The authors declare that they have no conflict of interest.

**Additional file 3**

**Supplementary figures**


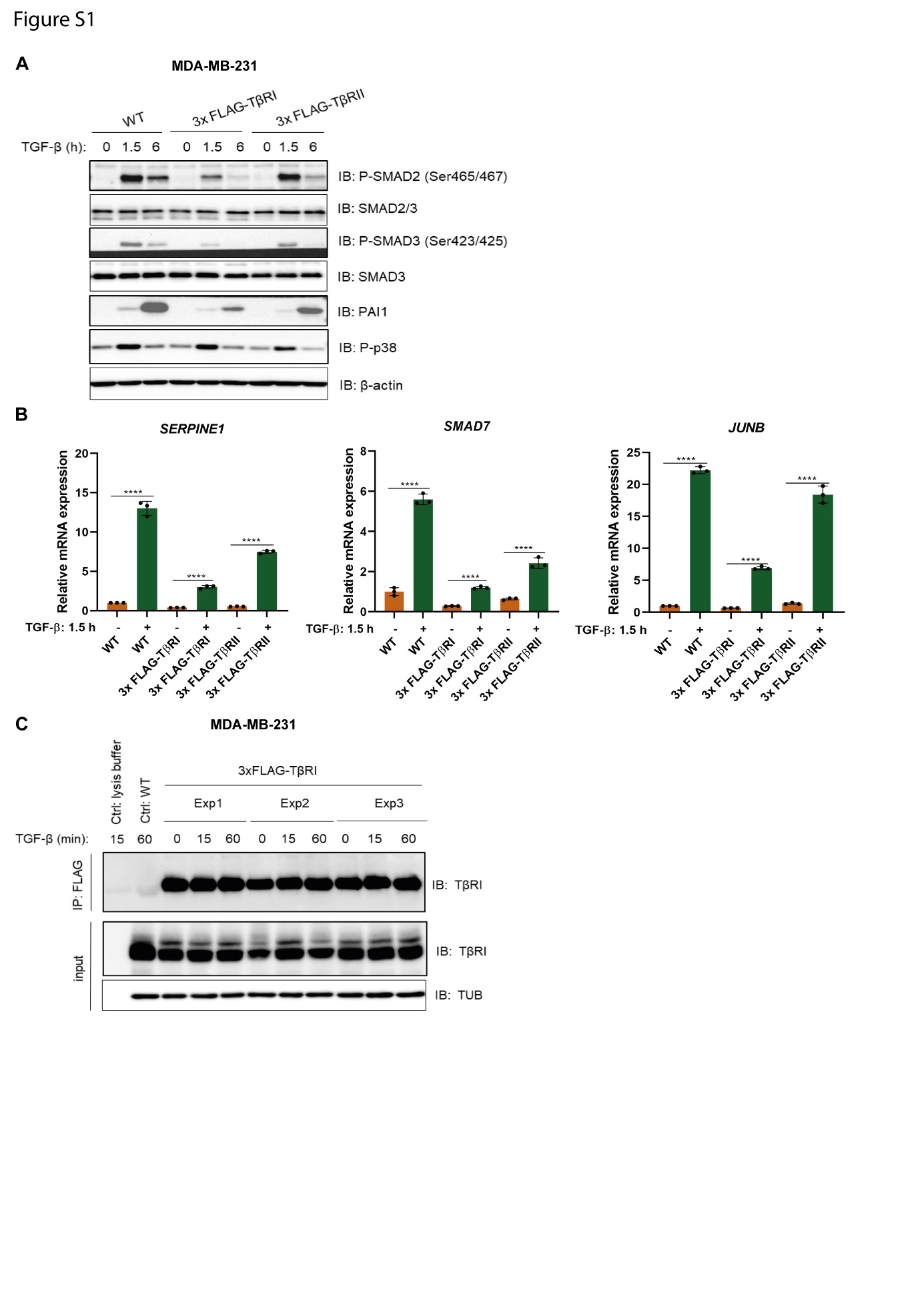


**Supplementary Figure 1 related to Main Figure 1**

Figure S1. **TGF-β signaling is preserved in MDA-MB-231 HiBiT-3×FLAG-TꞵRI cells, used for a mass spectrometry screen for TβRI binders.** (**A**) MDA-MB-231 WT cells, -HiBiT-3×FLAG-TꞵRI cells and -HiBiT-3×FLAG-TβRII cells were starved in DMEM, supplemented with 0.2% FBS, and treated or not with TGF-β (5 ng/ml) for 1.5 and 6 h. Total cell lysates were subjected to IB with the indicated antibodies. PAI1 (plasminogen activator inhibitor 1, encoded by *SERPINE1*) represents an early target gene of TGF-β signaling, and p-p38 MAPK represents non-SMAD signaling. (**B**) Total RNA from different cell lines was prepared, reversed to cDNA, and relative expression of *SERPINE1, SMAD7* and *JUNB* was examined by qRT-PCR and normalized to *GAPDH*. Data are presented in panel B as mean ± SD of three independent experiments; statistical significance was assessed by two-tailed unpaired Student’s t-tests. ****, p< 0.0001. (**C**) Validation of co-IP of TβRI for mass spectrometry screen. MDA-MB-231 HiBiT-3×FLAG-TꞵRI cells were starved and then stimulated or not with TGF-β (5 ng/ml) for 15 min and 60 min, in triplicates.


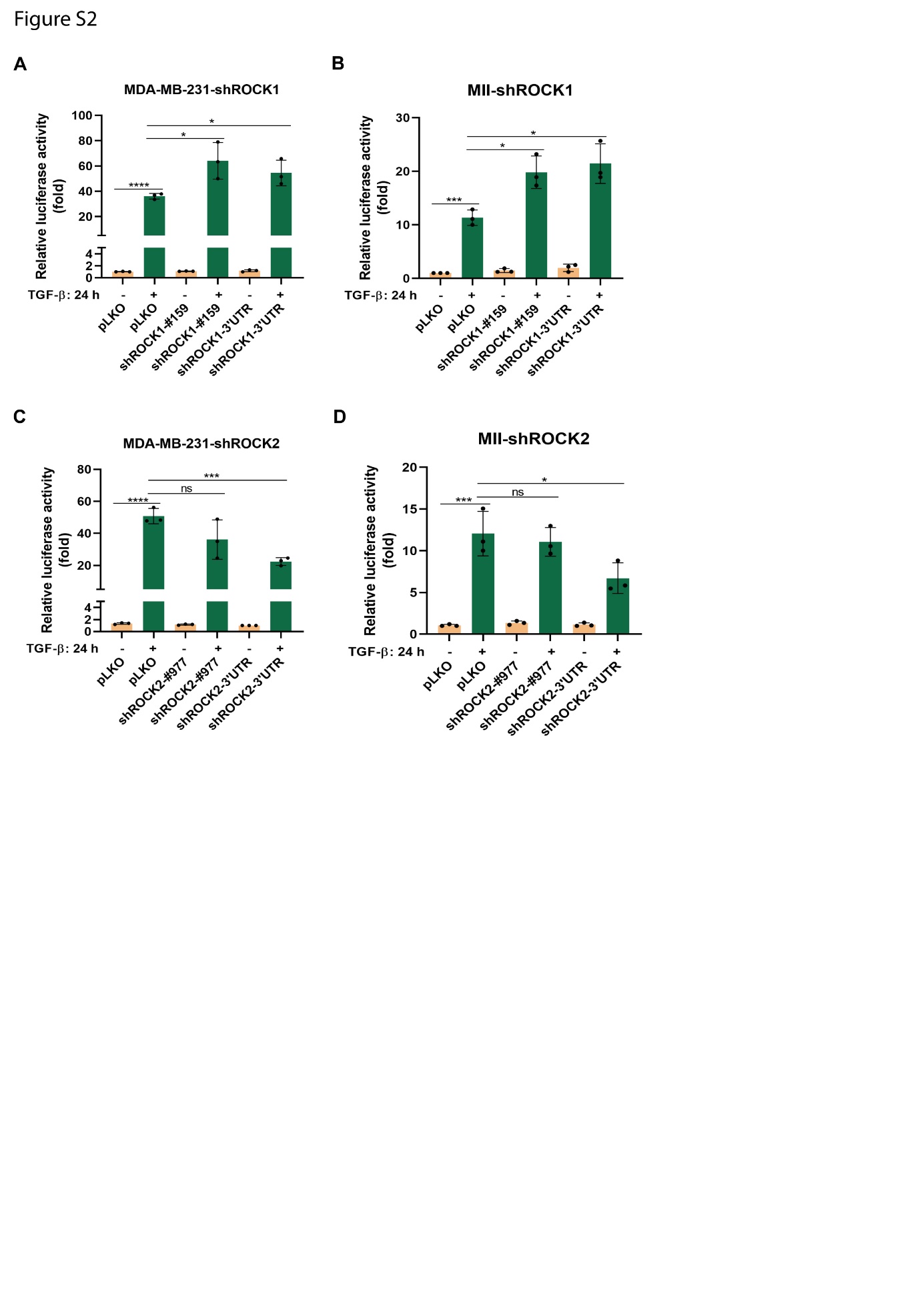


**Supplementary Figure 2 related to Main Figure 2**

Figure S2. **ROCK isoforms have opposite effects on TGF-β-induced activation of the CAGA_12_-Luciferase reporter.** **(A-D)** MDA-MB-231-pLKO and -shROCK1 cells **(A)**, MCF 10A MII-pLKO and- shROCK1 cells **(B)**, MDA-MB-231-pLKO and -shROCK2 cells **(C)**, and MCF 10A MII-pLKO and -shROCK2 cells (**D**), were transfected with CAGA_12_-luc and β-gal plasmids, starved and stimulated or not with TGF-β (1 ng/ml) for 24 h. The luciferase activities were measured by a Firefly Luciferase Assay Kit. Data are presented in panels A-D as mean ± SD of three independent experiments, statistical significance was assessed by two-tailed unpaired Student’s t-tests. ns, not significant difference; *, p<0.05; ***, p< 0.001; ****, p< 0.0001.


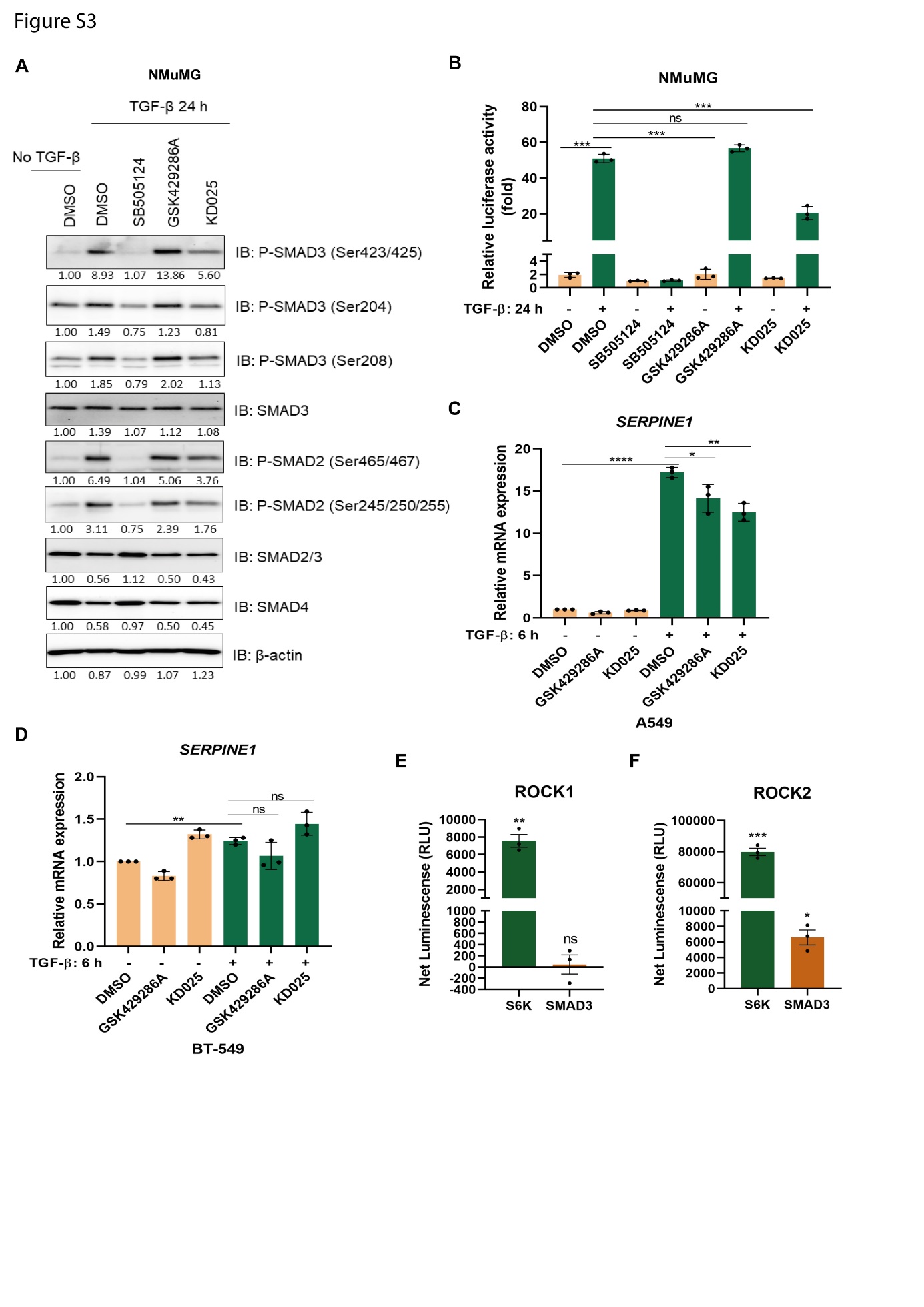


**Supplementary Figure 3 related to Main Figure 3**

Figure S3. **ROCK2 inhibition suppresses TGF-β-SMAD activity. (A)** NMuMG cells were starved in DMEM, supplemented with 0.2% FBS, treated or not with SB505124 (2.5 μM), GSK429286A (10 μM), or KD025 (5 μM) for 30 min, and then incubated with or without TGF-β (5 ng/ml) for 24 h. Total cell lysates were subjected to IB with the indicated antibodies. The band intensities were quantified and normalized to the DMSO condition set to 1.00, and further normalized to β-actin, by Image Lab software. (**B**) NMuMG cells transfected with CAGA_12_-luc and β-gal plasmids, were starved and supplemented with 0.2% FBS, treated or not with SB505124 (2.5 μM), GSK429286A (10 μM), KD025 (5 μM) and TGF-β (1 ng/ml) for 24 h. The luciferase activities were measured by a Firefly Luciferase Assay Kit. (**C**, **D**) Total RNA from A549 cells (**C**) and BT-549 cells (**D**) was prepared and reversed to cDNA, the relative expression of *SERPINE1* was examined by qRT-PCR and normalized to *GAPDH*. Data are presented in panel B-D as mean ± SD of three independent experiments, statistical significance was assessed by two-tailed unpaired Student’s t-tests. ns, not significant difference; *, p<0.05; **, p< 0.01; ***, p< 0.001; ****, p< 0.0001. (**E**, **F**) ROCK-mediated-SMAD3 phosphorylation. Recombinant ROCK1 or ROCK2 (10 ng) was incubated with S6K peptide (0.2 mg/ml) or SMAD3 (1 µM) in the presence of 25 µM ATP for 30 min, and the kinase activity was measured using the ADP-Glo assay kit. Data are shown as background-subtracted net phosphorylation, calculated as (ROCK + SMAD3 + ATP) − (ROCK + ATP) − (SMAD3 + ATP), and presented as mean ± SEM from three independent experiments. Statistical significance was assessed by one-sample t and Wilcoxon test versus zero. ns, not significant difference; *, p<0.05; **, p< 0.01; ***, p< 0.001.


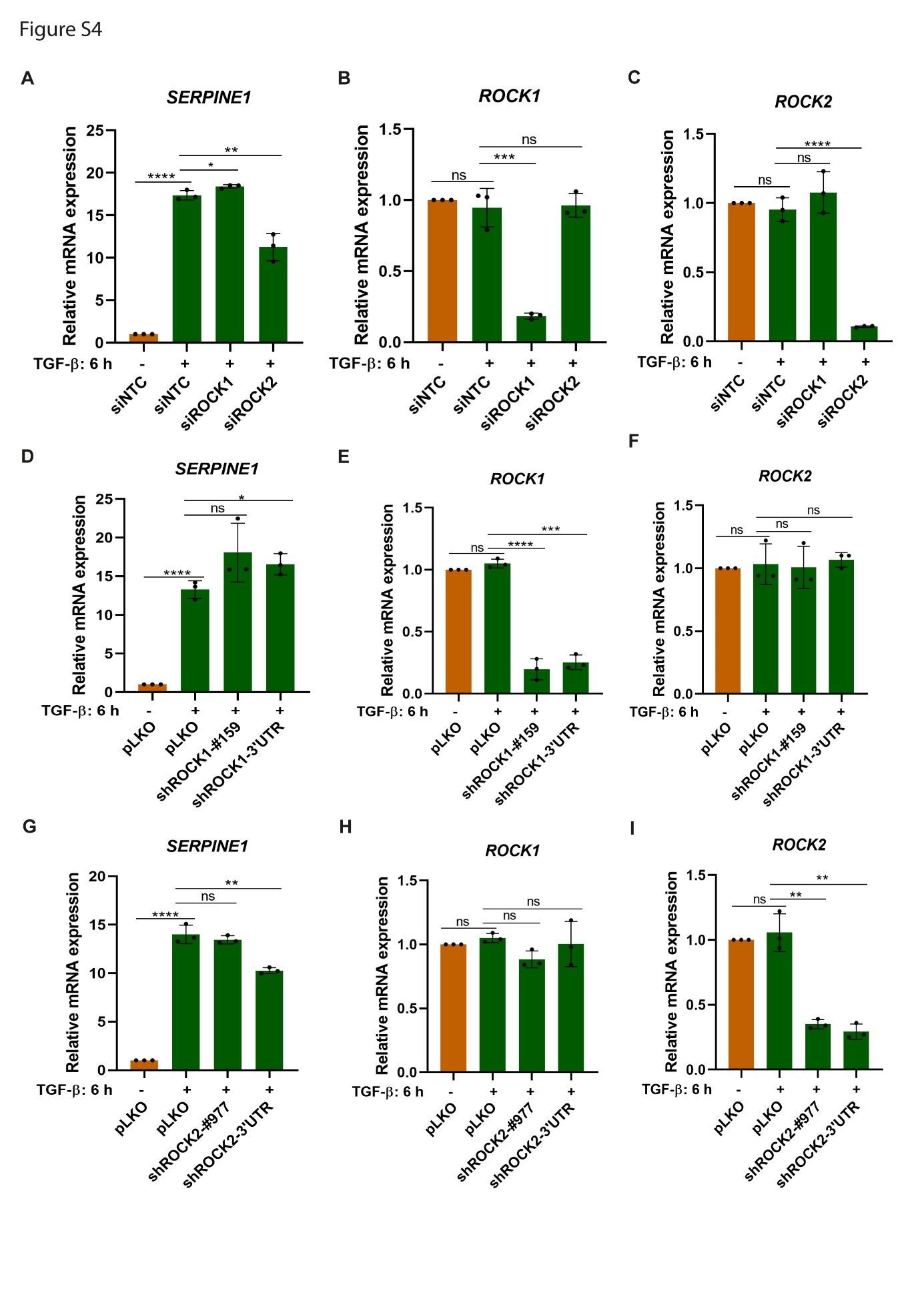


**Supplementary Figure 4 related to Main Figure 4**

Figure S4. **The effects of ROCK isoform depletion on TGF-β-SMAD target gene expression, and validation of knockdown efficiencies of ROCK isoforms**. MDA-MB-231 cells transfected with siControl (siNTC), siROCK1, siROCK2 (**A**, **B** and **C**), MDA-MB-231-pLKO and -shROCK1 cells (**D**, **E** and **F**), and MDA-MB-231-pLKO and -shROCK2 cells (**G**, **H** and **I**), were incubated with or without TGF-β (5 ng/ml) for 6 h. Expression of *SERPINE1* (A, D and G), *ROCK1* (B, E and H), *ROCK2* (C, F and I) was examined by RT-qPCR and normalized to *GAPDH*. Data are presented in panels A-I as mean ± SD of three independent experiments, statistical significance was assessed by two-tailed unpaired Student’s t-tests. ns, not significant difference; *, p<0.05; **, p< 0.01; ***, p< 0.001; ****, p< 0.0001.


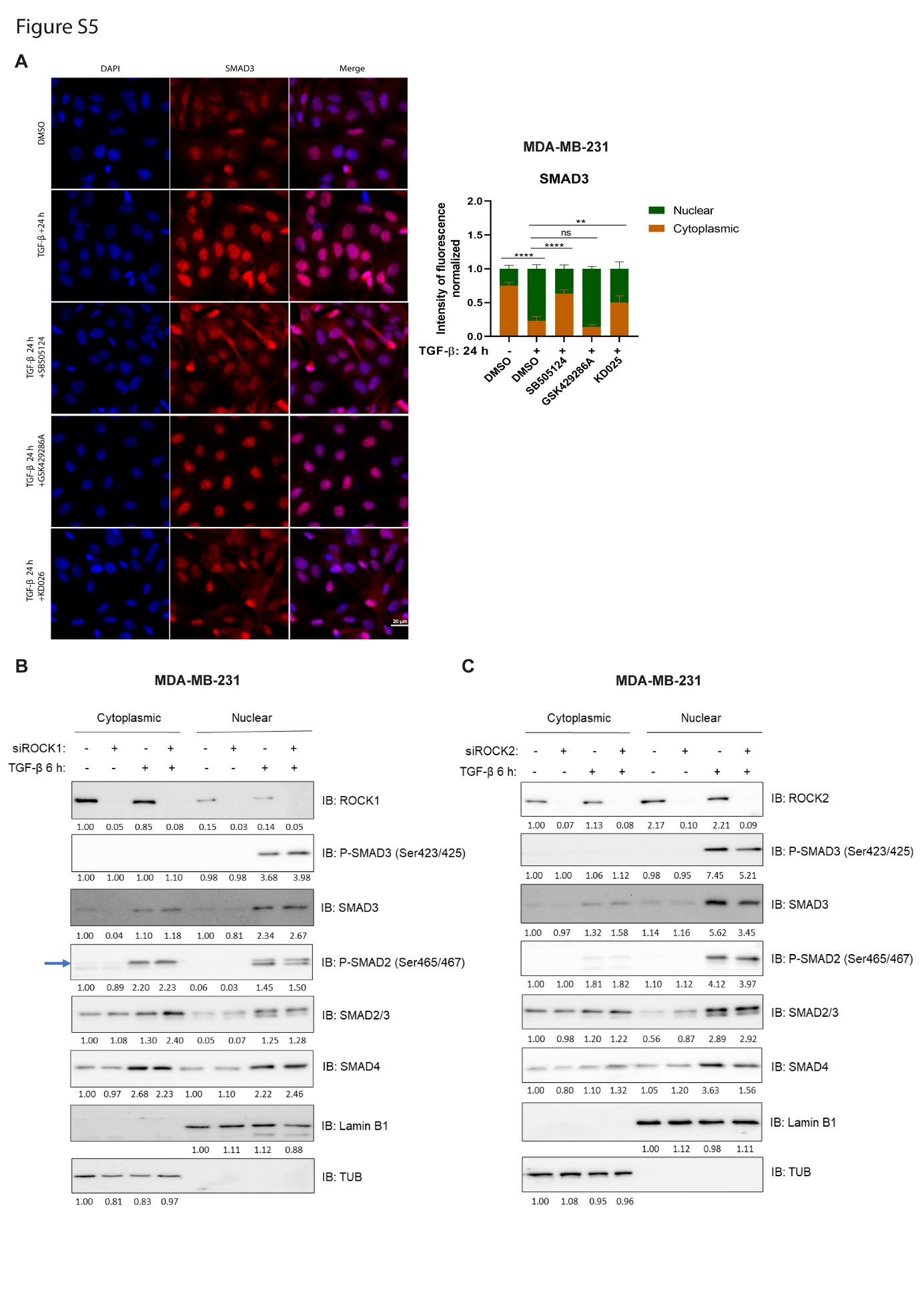


**Supplementary Figure 5 related to Main Figure 5**

Figure S5. **ROCK2 inhibition prevents SMAD3 nuclear localization.** (**A**) MDA-MB-231 cells were subjected to immunofluorescence staining using an antibody against SMAD3; nuclei were visualized by DAPI staining. The intensity of fluorescence in cytoplasmic and nuclear fractionations was quantified by Fiji (Image J) software. Scale bar indicates 20 µm. Quantification of data are presented in the right panel as mean ± SEM from three biological replicates, statistical significance was assessed by two-way ANOVA followed by Dunnett’s multiple comparisons test. ns, not significant difference; **, p< 0.01; ****, p< 0.0001. (**B-C**) Cytoplasmic and nuclear fractionations of MDA-MB-231 cells transfected with siControl (siNTC), siROCK1 (**B**), siROCK2 (**C**), and treated or not with TGF-β (5 ng/ml), were prepared and subjected to IB with the indicated antibodies. The band intensities were quantified and normalized to the cytoplasmic control condition set 1.00, and further normalized to Tubulin (TUB) or Lamin B1, by Image Lab software.


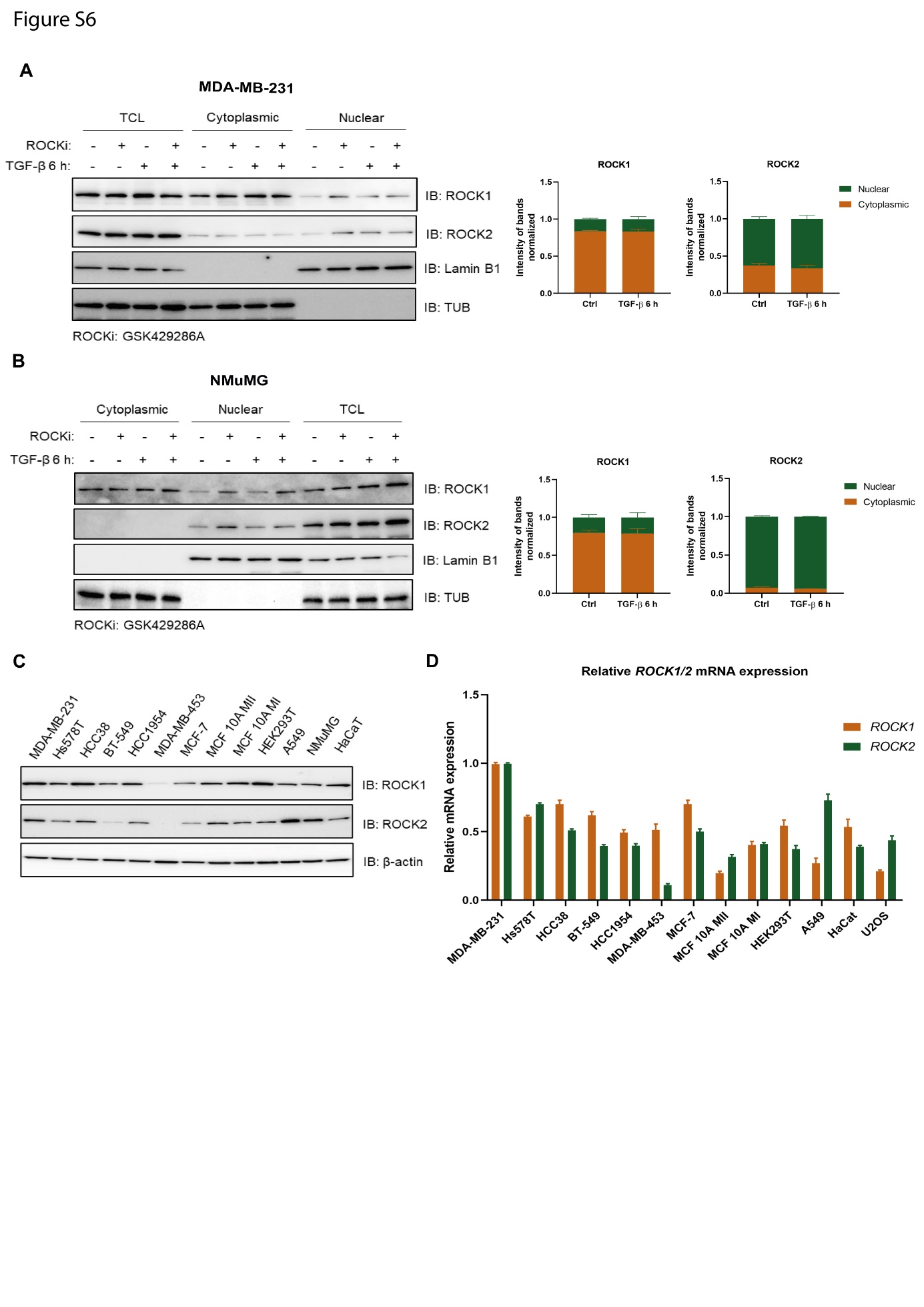


**Supplementary Figure 6 related to Main Figure 6**

Figure S6. **ROCK1 and ROCK2 exhibit distinct subcellular localizations, and the expression of ROCK1 and ROCK2 protein and mRNA varies across cell lines.** (**A**, **B**) Cytoplasmic and nuclear fractions of MDA-MB-231 (**A**) and NMuMG (**B**) cells, treated or not with TGF-β (5 ng/ml) and GSK429286A (10 μM), were prepared and subjected to IB with the indicated antibodies. The intensities of bands in each fraction were quantified and normalized to Tubulin (TUB; cytoplasmic fraction) or Lamin B1 (nuclear fraction), by the Image Lab software. (**C**) Total cell lysates from different cell lines were subjected to IB using antibodies against ROCK1 and ROCK2. The intensities of bands were quantified and normalized to the first cell line (MDA-MB-231) set to 1.00, and further normalized to β-actin, by the Image Lab software. (**D**) Total RNA from different cell lines were prepared and reversed to cDNA; the relative gene expression was then determined by RT-qPCR and normalized to *GAPDH*.
