# Supplementary material for: Opposing effects of Rho-associated coiled-coil kinase 1 (ROCK1) and ROCK2 in TGF-β-SMAD signaling
Source: Cell Commun Signal. 2026 Feb 7;24:137. doi: 10.1186/s12964-026-02722-5 (PMC12930806; doi:10.1186/s12964-026-02722-5)
Supplement: Supplementary file 4 — Additional file 4. Uncropped immunoblots [file 12964_2026_2722_MOESM4_ESM.docx]

**Opposing effects of Rho-associated coiled-coil kinase 1 (ROCK1) and ROCK2**

**in TGF-β-SMAD signaling**

Yu Bai^1,2^*, Mohamad Moustafa Ali^1^, Maarten van Dinther^3^, Peter ten Dijke^3^, Aristidis Moustakas^1^, Anders Sundqvist^1,4^ and Carl-Henrik Heldin^1^*

^1^Department of Medical Biochemistry and Microbiology, Science for Life Laboratory, Box 582, Biomedical Center, Uppsala University, SE-75123 Uppsala, Sweden

^2^Present address: Department of Immunology, Genetics and Pathology, Science for Life Laboratory, Uppsala University, SE-751 85 Uppsala, Sweden

^3^Department of Cell and Chemical Biology, Oncode Institute, Leiden University Medical Center, Leiden, The Netherlands

^4^Department of Pharmaceutical Biosciences, Uppsala University, Sweden

**Running title**: Opposing Roles of ROCK1 and ROCK2 in TGF-β Signaling

*Corresponding authors: Yu Bai, Department of Immunology, Genetics and Pathology, Science for Life Laboratory, Uppsala University, SE-751 85 Uppsala, Sweden. E-mail: yu.bai@igp.uu.se

Carl-Henrik Heldin, Department of Medical Biochemistry and Microbiology, Science for Life Laboratory, Box 582, Biomedical Center, Uppsala University, SE-75123 Uppsala, Sweden. E-mail: c-h.heldin@imbim.uu.se

**Conflict of interest**: The authors declare that they have no conflict of interest.

**Original immunoblots**

Uncropped immunoblots determining the expression of the indicated proteins with molecular size markers. The cropped region presented in the main and supplementary figures were marked with dotted rectangles.

**
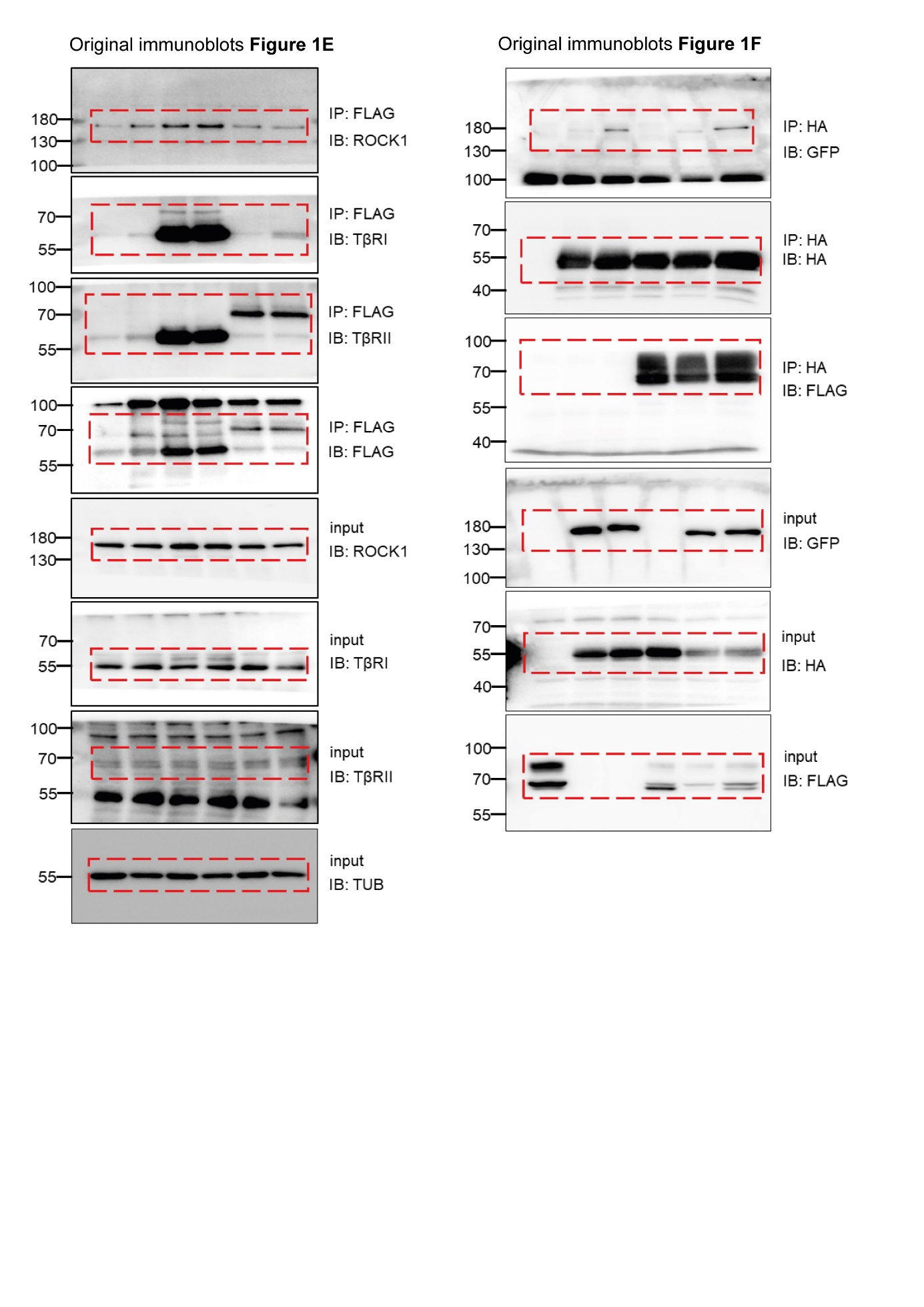
**

**
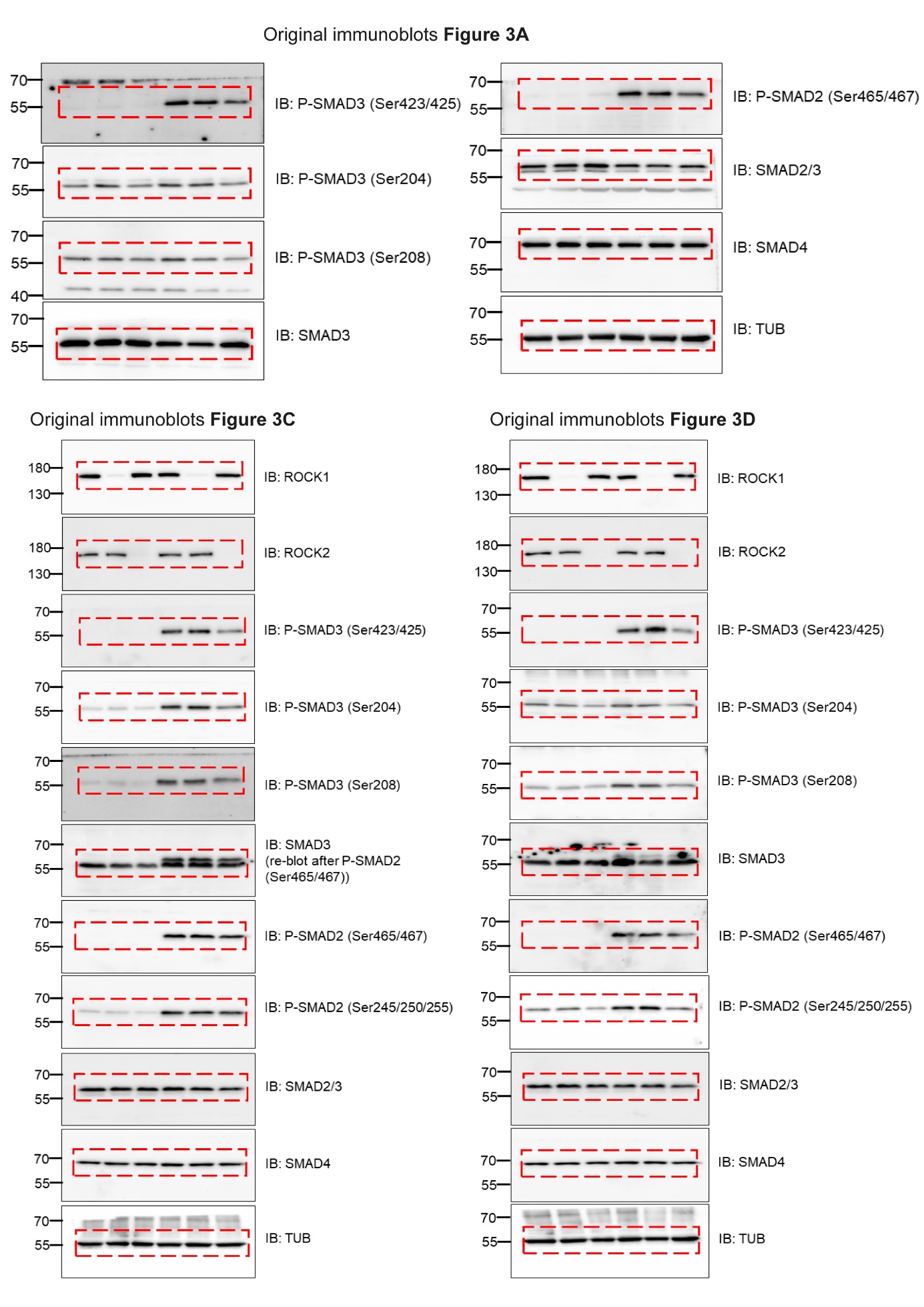
**

**
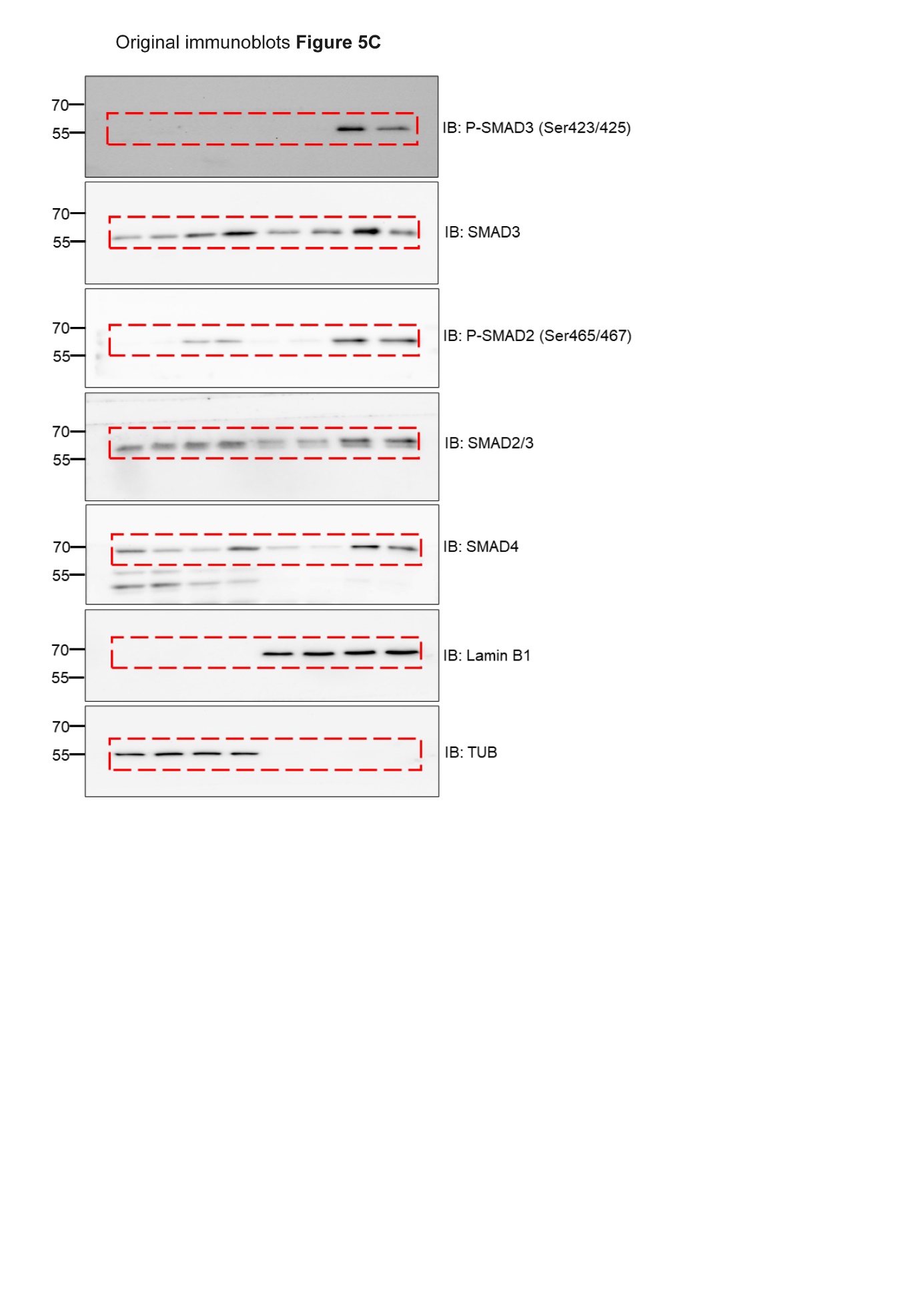
**

**
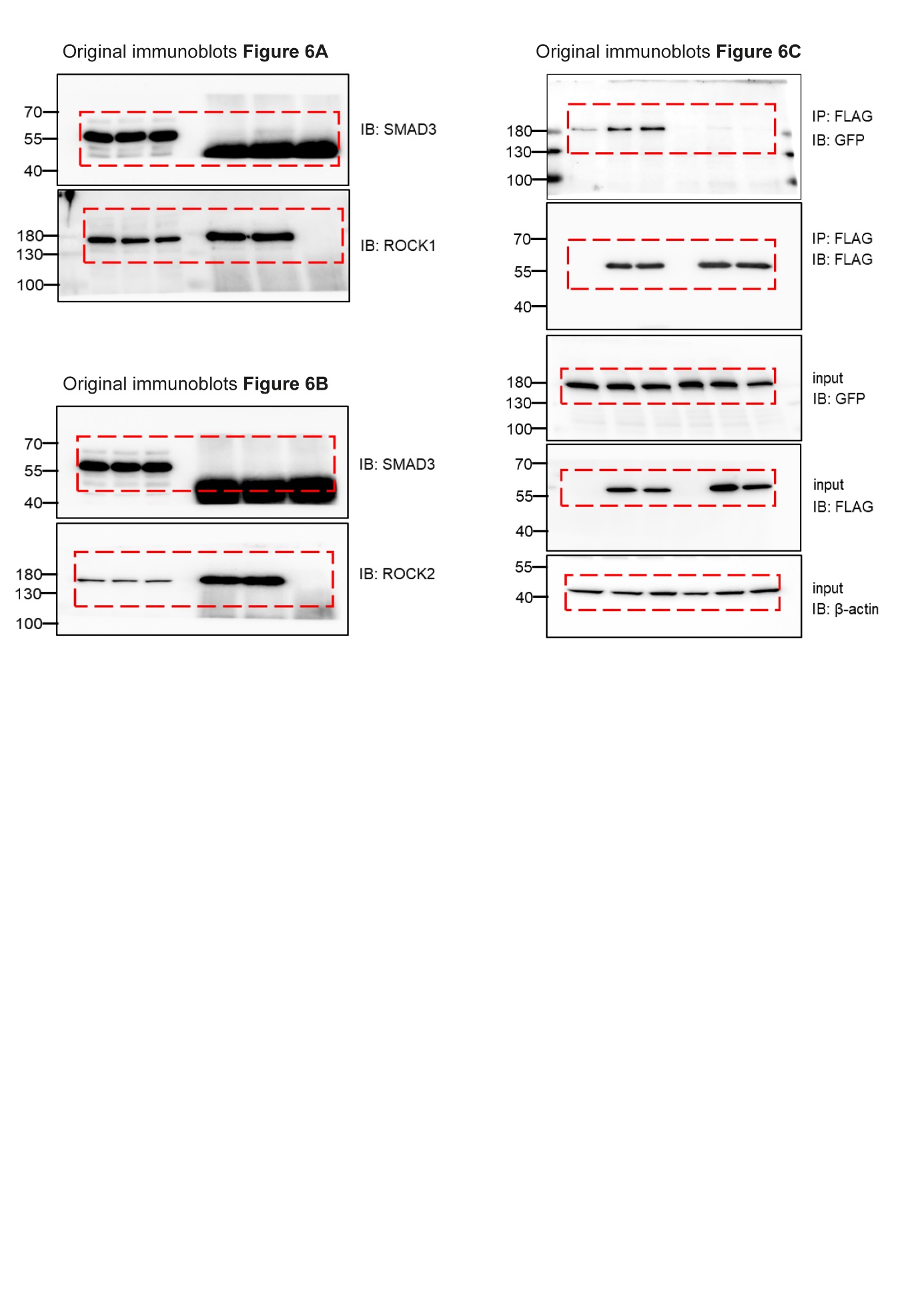
**

**
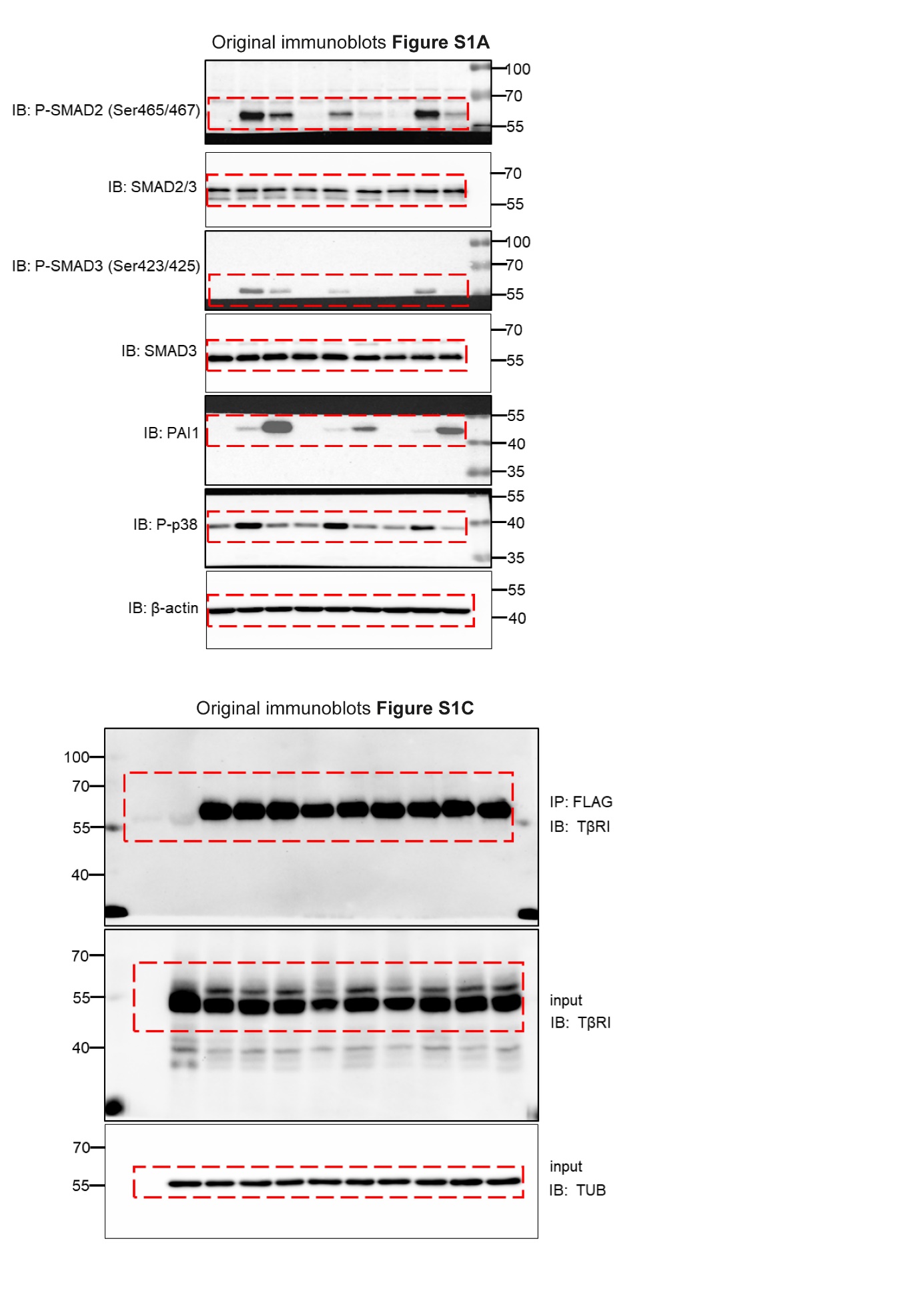
**

**
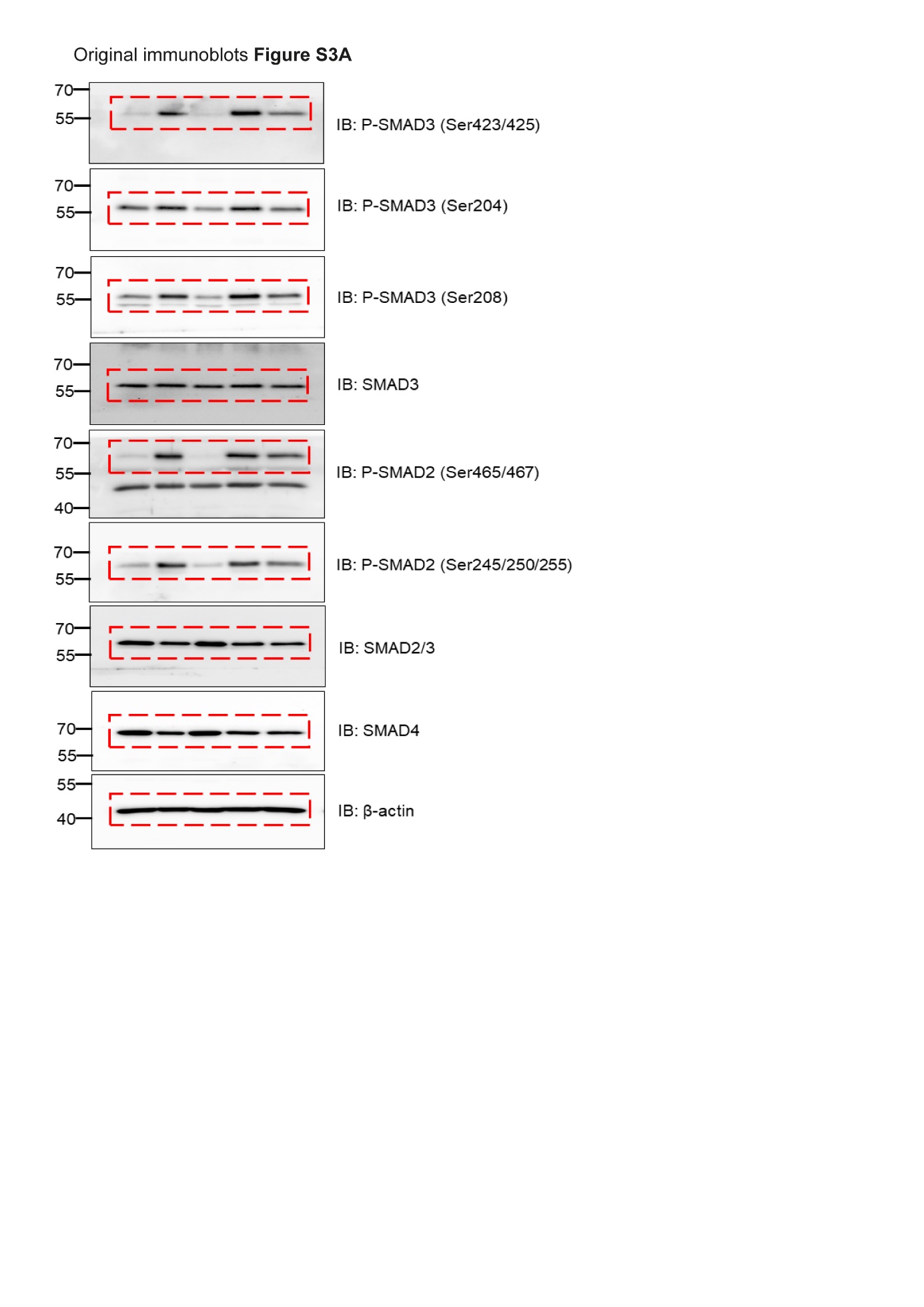
**

**
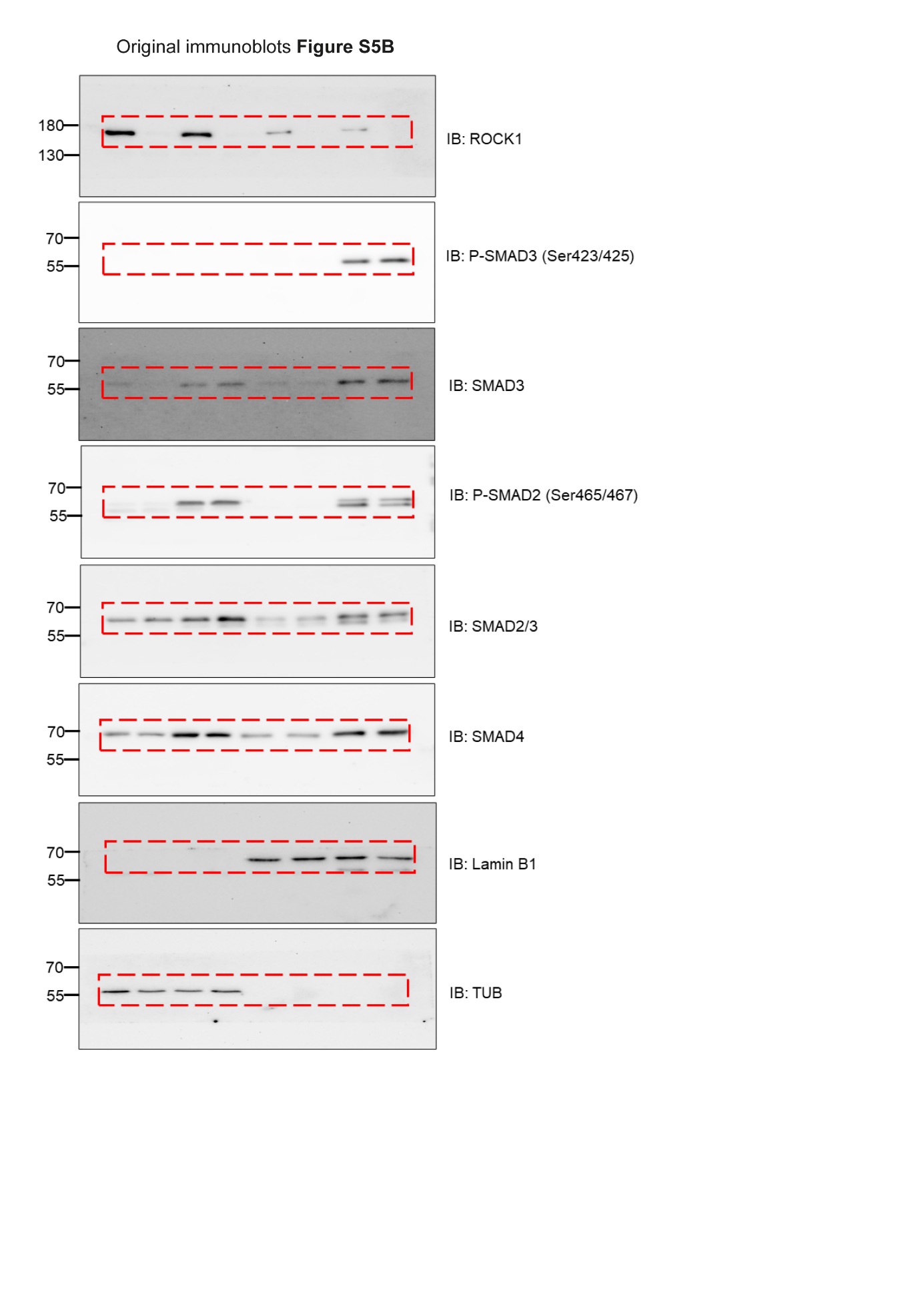
**

**
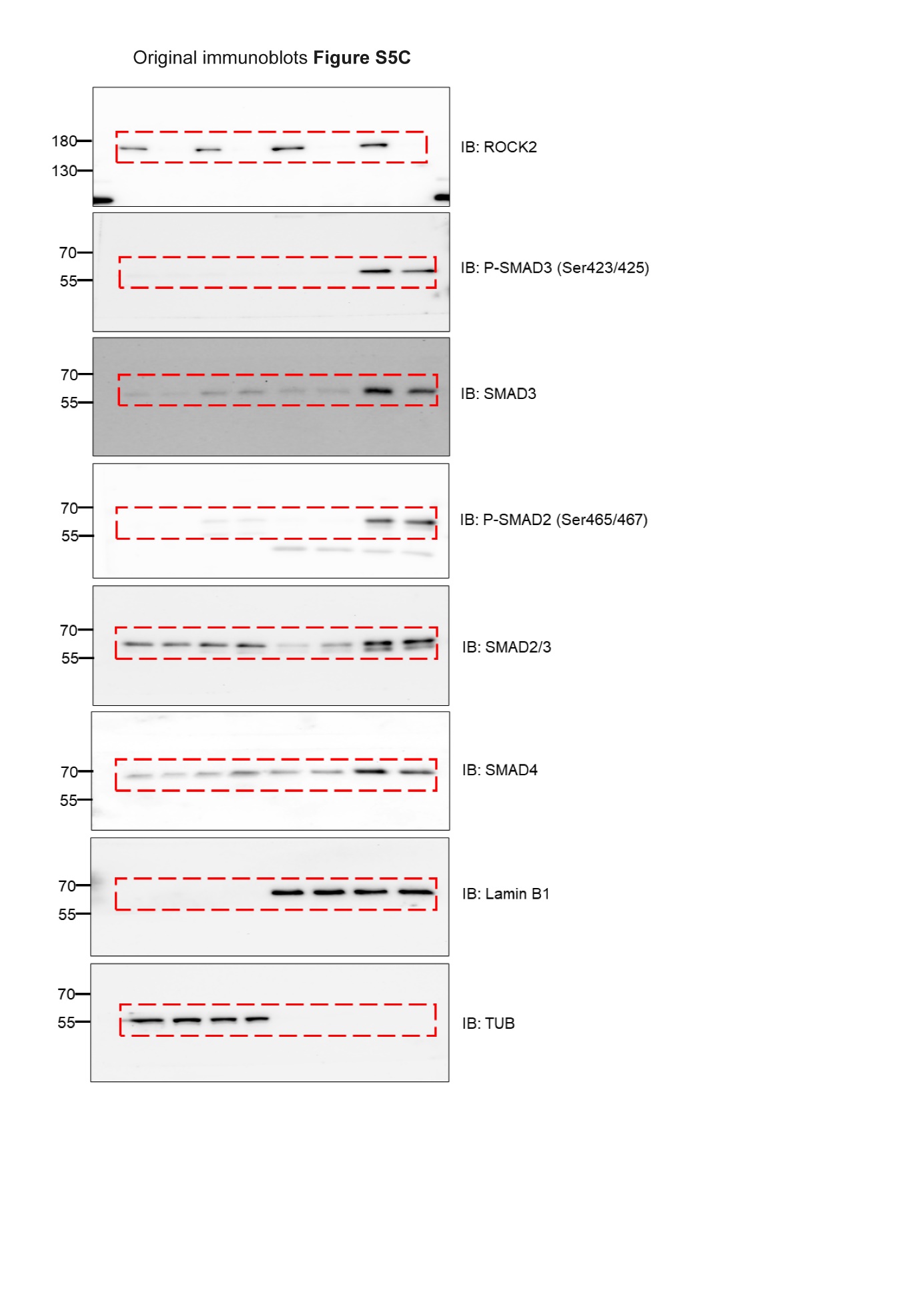
**

**
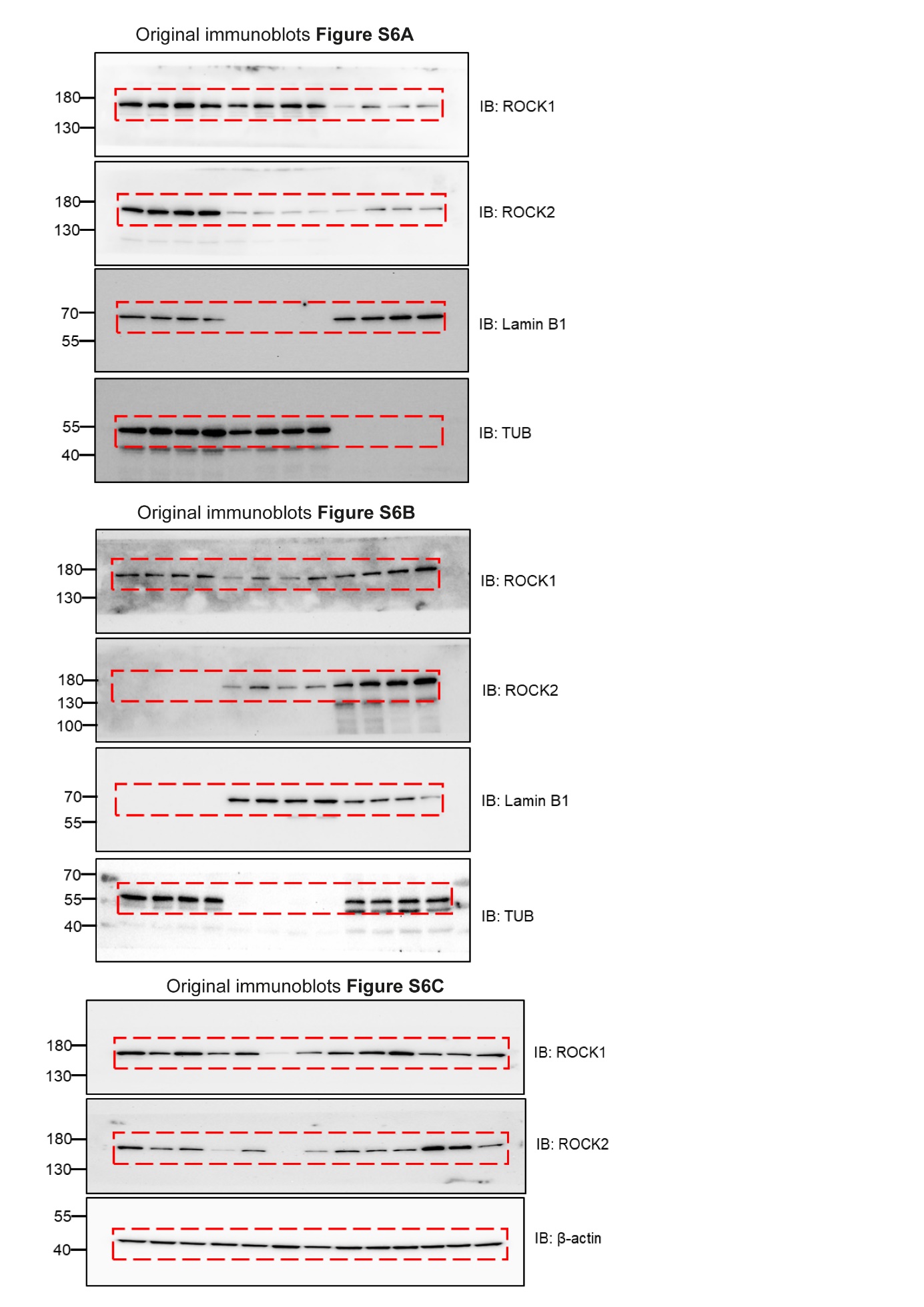
**
